# Inference of the Demographic Histories and Selective Effects of Human Gut Commensal Microbiota Over the Course of Human History

**DOI:** 10.1093/molbev/msaf010

**Published:** 2025-01-22

**Authors:** Jonathan C Mah, Kirk E Lohmueller, Nandita R Garud

**Affiliations:** Bioinformatics Interdepartmental Program, University of California, Los Angeles, USA; Department of Ecology and Evolutionary Biology, University of California, Los Angeles, USA; Department of Human Genetics, University of California, Los Angeles, USA; Department of Ecology and Evolutionary Biology, University of California, Los Angeles, USA; Department of Human Genetics, University of California, Los Angeles, USA

**Keywords:** microbiome, DFE, selection, population genetics, demography, evolution

## Abstract

Despite the importance of gut commensal microbiota to human health, there is little knowledge about their evolutionary histories, including their demographic histories and distributions of fitness effects (DFEs) of mutations. Here, we infer the demographic histories and DFEs for amino acid-changing mutations of 39 of the most prevalent and abundant commensal gut microbial species found in Westernized individuals over timescales exceeding human generations. Some species display contractions in population size and others expansions, with several of these events coinciding with several key historical moments in human history. DFEs across species vary from highly to mildly deleterious, with differences between accessory and core gene DFEs largely driven by genetic drift. Within genera, DFEs tend to be more congruent, reflective of underlying phylogenetic relationships. Together, these findings suggest that gut microbes have distinct demographic and selective histories.

## Introduction

Diversity in the human gut microbiome varies significantly across geographic regions around the world. For example, rural African microbiomes display greater levels of diversity than urban Western microbiomes at multiple levels including at the species (Sonnenburg et al. 2016), gene (Tett et al. 2019), and nucleotide (Nayfach and Pollard 2015; Tett et al. 2019) levels. A number of demographic and selective forces may be responsible for these differences in diversity, including urbanization (Obregon-Tito et al. 2015), antibiotic usage (Clemente et al. 2015; Sonnenburg and Sonnenburg 2019), shifts in diet (Filippo et al. 2010; Schnorr et al. 2014; Blaser 2018), and human migration (Yatsunenko et al. 2012). For example, reductions in dietary fiber in mice lead to irreversible reductions in genetic diversity in the gut microbiome and are thought to be a contributing factor to the depleted diversity of rural agrarian populations compared to hunter–gatherer populations (Sonnenburg et al. 2016). Additionally, some microbes such as *Helicobacter pylori* (Falush et al. 2003) and *Prevotella copri* (Tett et al. 2019) display phylogenetic patterns mirroring human migration events around the world, suggesting that human demographic events themselves may have shaped bacterial genomic variation.

To date, there has not been a systematic inference of the demographic histories and distributions of fitness effects (DFEs) of new mutations of human commensal gut microbiota, nor has there been any investigation into how these quantities might vary across species. Such inferences are important for a number of reasons. Demographic inference provides an understanding of pre-historical events, such as continental migrations as well as population contractions and expansions. Additionally, demographic inference enables the ability to detect genomic regions subject to selective forces by providing an expectation of genetic diversity under neutral conditions. Inference of the DFE, which quantifies the proportion of new mutations that are deleterious, neutral, or beneficial, is necessary for addressing fundamental questions such as the fate of colonizing lineages within a human host and the evolutionary capacity of a population to respond to novel selection pressures (Chevin et al. 2010; Dapa et al. 2023).

**Fig. 1.**
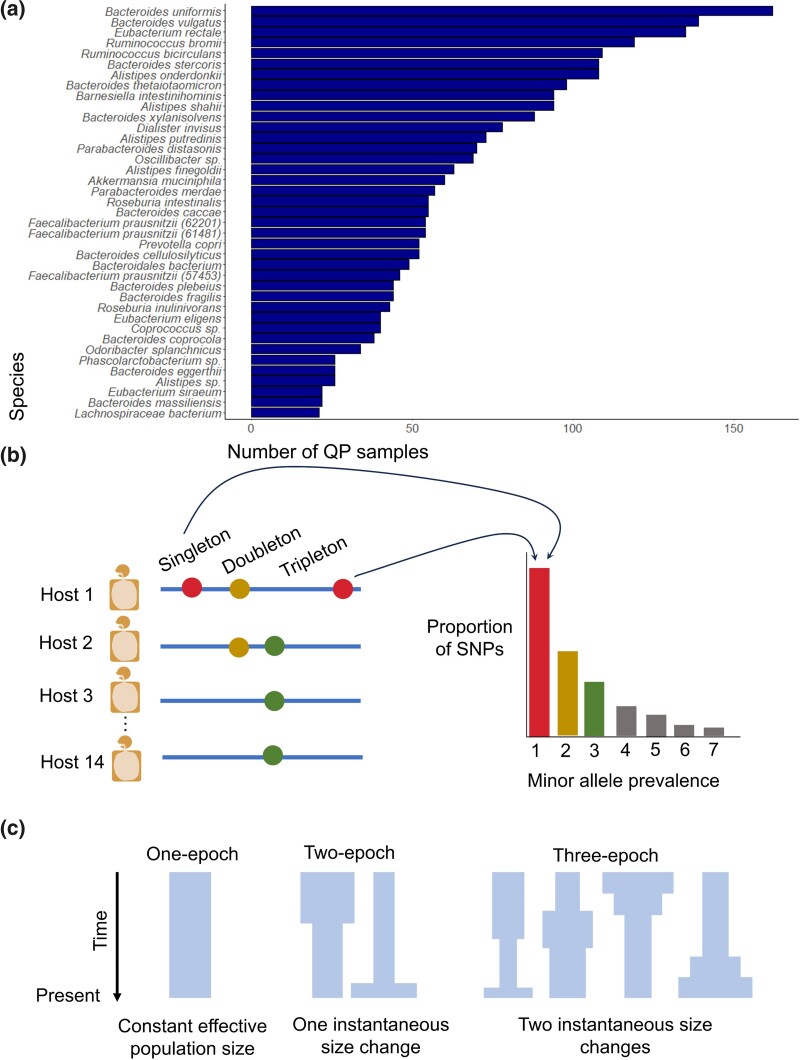
Data and schematic of putative demographic models. a) Number of quasi-phaseable (QP) samples for the 39 most prevalent species in the datasets analyzed. b) A single quasi-phased haplotype was inferred for each species for each host with that species. Shown are four example quasi-phased haplotypes. These haplotypes harbor SNPs that are either “singletons” (present in a single host), “doubletons”, “tripletons”, etc. SFSs were then constructed by binning the prevalences of alleles found across the quasi-phaseable haplotypes for each species. c) SFSs were then used to infer parameters for one-, two-, and three-epoch models, as depicted. Time is shown on the *y*-axis. The width of each model represents effective population size at the corresponding time. A one-epoch model has a constant effective population size and can be considered to be a null model against any models which permit effective population size changes. A two-epoch model has a single instantaneous change in effective population size, allowing for inference of a contraction or expansion. A three-epoch model has two instantaneous changes in effective population size, allowing for inference of multiple contractions and/or expansions.

A key population genetics approach (Wakeley and Hey 1997; Nielsen 2000; Gutenkunst et al. 2009) for the inference of demographic histories and DFEs among eukaryotes leverages a summary statistic known as the site-frequency spectrum (SFS) (Fig. 1b). The SFS describes the distribution of minor allele frequencies from a given sample of DNA sequences and is highly sensitive to the impact of demography and selection (Nielsen 2000). For example, relative to the SFS of a population in demographic equilibrium, a population undergoing a demographic expansion and/or experiencing purifying selection would harbor a higher proportion of rare variants, i.e. single nucleotide polymorphisms (SNPs) present in a small number of individuals relative to the overall sample. Conversely, a population undergoing a demographic contraction and/or experiencing balancing selection would harbor a higher proportion of intermediate frequency variants.

While the SFS has been extensively used to infer demography and selective effects in eukaryotes (Eyre-Walker et al. 2006; Boyko et al. 2008; Gutenkunst et al. 2009; Beichman et al. 2018), it has been under-utilized for bacteria (Cornejo et al. 2013; Pepperell et al. 2013; Mortimer et al. 2017). Yet, with this approach, the cavity-causing oral bacterium *Streptococcus mutans* was inferred to have undergone a demographic expansion, coincident with the onset of agriculture ∼10,000 to 20,000 years ago (Cornejo et al. 2013), potentially due to an expanded niche generated by shifts in diet. Additionally, mutations in this species, like those in many other bacterial species, were inferred to experience extensive purifying selection (Cornejo et al. 2013). Application of SFS-based analyses to human gut commensal bacteria may similarly yield insights into demographic events and DFEs of new mutations.

One important assumption of an SFS-based analysis is quasi-independence between genomic loci, i.e. free recombination (Bustamante et al. 2001; Lyulina et al. 2024). While bacteria reproduce asexually, most commensal gut bacteria experience extensive recombination (Smith et al. 1993; Vos and Didelot 2009; Garud et al. 2019; Lin and Kussell 2019; Sakoparnig et al. 2021; Liu and Good 2024). This recombination plays a critical role in shuffling genetic material and breaking up correlations between loci in the genome on long timescales exceeding within-host evolution. The effects of recombination on bacterial genomic diversity is evident in the rapid decay in linkage disequilibrium (LD) with genomic distance in samples of lineages from human hosts around the world (Garud et al. 2019; Lin and Kussell 2019; Sakoparnig et al. 2021; Liu and Good 2024), as well as phylogenetic inconsistencies at the SNP and gene level with trees built from genome-wide data (Garud et al. 2019, Sakoparnig et al. 2021). It has been estimated that recombination contributes on the order of 10 times more to variation than mutations to human commensal gut bacteria, indicating that recombination overwrites any semblance of clonality among circulating lineages (Liu and Good 2024). Given the relatively high rates of recombination in gut microbiota, we propose to use the SFS constructed using a collection of diverse lineages *across* hosts to gain insights into the evolutionary history of commensal bacteria.

In this study, we perform an SFS-based inference of the demographic history and fitness effects acting on nonsynonymous mutations in 39 prevalent commensal gut species using a panel of healthy hosts from North America. Concretely, we examine the evolutionary dynamics operating across hosts on timescales exceeding a human lifetime by building SFSs composed of a single strain per individual microbiome for each species. We infer that human gut commensal bacteria have experienced a range of demographic histories, including contractions and expansions in effective population size over the past ∼10^3^ to 10^6^ years of human history. Additionally, we infer DFEs from the SFS, and find that bacterial DFEs are more congruent within versus between-genera, indicative of underlying phylogenetic relationships. Finally, we find that both genetic drift and natural selection play a role in driving differences in the DFE between core and accessory genes, with drift likely being a stronger driving force. Taken together, these findings suggest that common commensal gut microbiota have distinct evolutionary histories.

## Results

### Data

To infer the demographic histories and DFEs of new mutations of gut commensal bacteria found in Western urbanized populations, we analyzed shotgun metagenomic data from a panel of 693 healthy hosts from four datasets from North America (Human Microbiome Consortium 2012; Lloyd-Price et al. 2017), Europe (Xie et al. 2016; Korpela et al. 2018), and China (Qin et al. 2010) that we previously analyzed (Garud et al. 2019). To identify SNPs and gene copy number variants (CNVs) from these metagenomic samples, reads were mapped to reference genomes using a standard pipeline (Nayfach et al. 2016) (See Methods). Since each host may be colonized by multiple strains of the same species, to obtain a sample of lineages across hosts, we leveraged a previous approach we developed to “quasi-phase” haplotypes corresponding to the dominant strain present in each host for each species (Garud et al. 2019) (See Methods). In total, we obtained 5,416 “quasi-phased” (QP) genomes from 43 different species across the hosts in our datasets (Fig. 1a). This collection of genomes provided the ability to infer the demographic histories of bacteria in Westernized individuals.

### Gut Microbiota Experience a Range of Demographic Changes on Time Scales Spanning Human History

#### Building SFSs From Metagenomic Data From a Panel of Hosts

To infer demographic histories of human gut commensal microbiota, we analyzed SFSs composed of lineages from different hosts as opposed to within-host SFSs. SFSs composed of diverse lineages across hosts may contain information about population histories on timescales exceeding a human lifetime as opposed to demographic changes occurring within a host (Fig. 1). Moreover, lineages found across hosts experience extensive levels of recombination, while lineages within a host may be derived from a recent clonal ancestor.

SFSs were composed of one QP genome per host for each of 39 of the most prevalent bacterial species that had a minimum of 14 QP genomes (See Methods; Fig. 1a). We required a minimum sample size of 14 QP genomes to allow for a large number of species to be included in our analyses, while maintaining enough bins in the SFS to infer population demographic histories. Additionally, we initially focused our analysis on the core genome of each species, i.e. genes present in at least 95% of samples (See Methods).

Since population structure can result in false inferences of demography and selection by generating an excess of intermediate frequency SNPs (Städler et al. 2009; Gazave et al. 2014), we controlled for population structure at two levels. First, while different hosts typically harbor their own genetically distinct set of strains, occasionally there are some exceptionally closely related lineages (divergence < 2 × 10^−4^/bp) circulating in the population (Garud et al. 2019). To avoid structure arising from potential clonal expansions, we excluded lineages with divergences < 2 × 10^−4^/bp to any other lineage in the dataset, as we previously did to control for population structure (Garud et al. 2019) (See Methods).

Second, previous work has found that several gut bacterial species comprise deeply diverged clades lacking gene flow between clades (Costea et al. 2017; Garud et al. 2019; Liu and Good 2024). In other words, lineages belonging to the same clade exhibit significantly higher amounts of recombination than lineages part of different clades, as demonstrated by more rapid rates of LD decay and increased phylogenetic inconsistencies of individual SNPs with a genome-wide phylogeny (Garud et al. 2019; Liu and Good 2024). Thus, to control for population structure as a potential confounder for demographic inference, we analyzed SFSs belonging to lineages comprising the largest clade for each species, based on our previous clade inferences (Garud et al. 2019). In supplementary fig. S1, Supplementary Material online we show the impact of population structure control and subsequent projection on the shape of the empirical SFS.

Finally, selective forces acting on nonsynonymous sites may confound inference of demography by changing the shape or skew of the SFS. For example, purifying selection typically results in an excess of rare variants, thus skewing the nonsynonymous SFS toward low frequency SNPs. By contrast, the synonymous SFS is thought to be more neutrally evolving, thereby allowing for more sensitive inference of the underlying demographic history (Williamson et al. 2005; Boyko et al. 2008). To control for the potential effects of selection, we constructed SFSs using only synonymous SNPs.

#### Demographic Inferences With ∂a∂i

To infer the demographic histories of gut commensals, we used a statistical inference package known as ∂a∂i (Gutenkunst et al. 2009), which is a maximum-likelihood inference method based on the SFS. We explored three different putative demographic models in this framework for each species, permitting the inference of population expansions versus contractions (Fig. 1c). Selection of the best-fitting model was performed using the Akaike information criterion (AIC) (See Methods; supplementary fig. S2, Supplementary Material online).

**Fig. 2.**
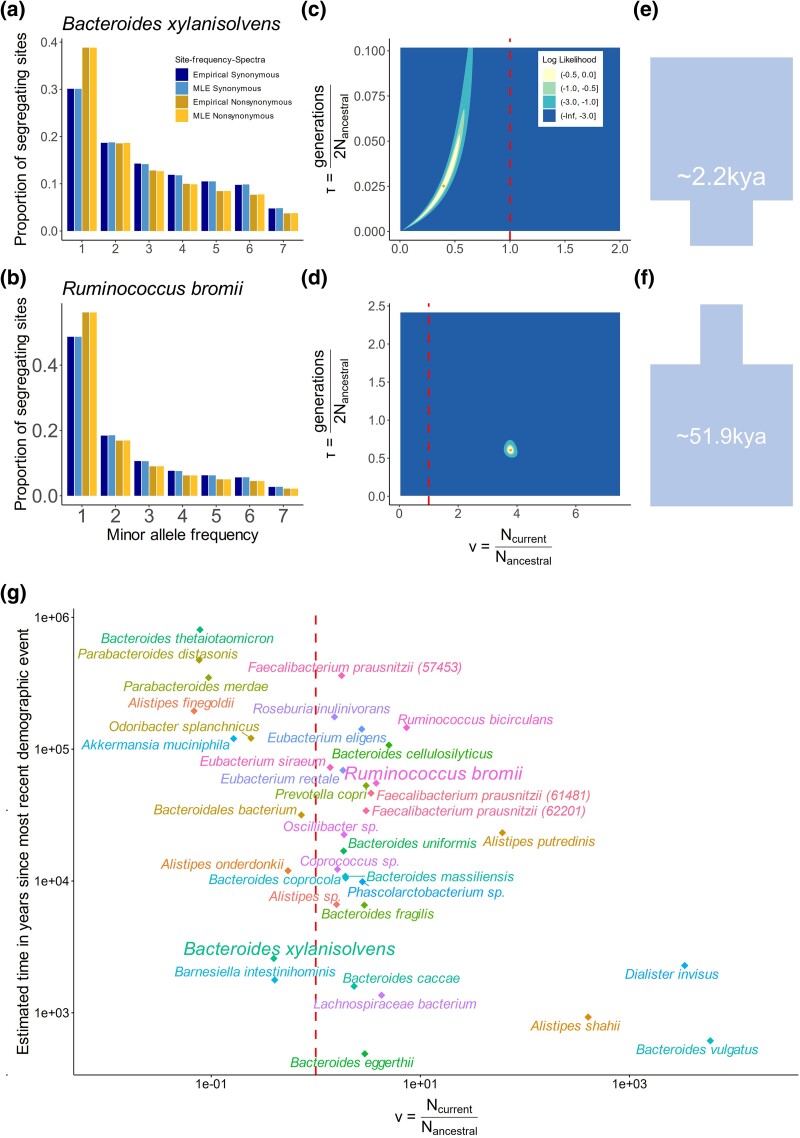
Demographic inference of common commensal gut microbes. Site-frequency spectra from the empirical data are compared to those predicted from the best-fit demographic models for two species: a) *Bacteroides xylanisolvens* and b) *Ruminococcus bromii. “*MLE synonymous” shows the expected SFS produced by the ML demographic parameter estimates. “MLE nonsynonymous” shows the expected SFS produced by the ML demographic and selection parameters from a gamma-distributed DFE. Two-dimensional likelihood surfaces of population size, *ν* (in units of *N*_Anc_) and time since the onset of the most recent demographic event, *τ* (in units of $\frac{generations}{2N_{Anc}\;}$) are shown for c) *B. xylanisolvens*, and d) *R. bromii*. A dashed red line separates the parameter space between contractions and expansions. The maximum-likelihood demographic parameters are shown with an orange dot, and colored intervals denote the decrease in log-likelihood from the MLE. The light cyan regions (LL—3) correspond to the asymptotic 95% confidence interval from the log-likelihood surface. See supplementary fig. S3, Supplementary Material online for similar plots for all species analyzed. Cartoon schematics (approximately to scale) describing the inferred demography of e) *B. xylanisolvens*, and f) *R. bromii*. g) A summarizing two-dimensional scatterplot of ν and approximate time in years for ML demographic parameters for the 36 of the 39 species analyzed in this paper which were best-fit to a two-epoch demographic model. There are three species named *Faecalibacterium prausnitzii*, and so their distinct species IDs as per the MIDAS (Nayfach et al. 2016) database used to map read are indicated in parentheses.

Demographic inference performed with ∂a∂i recovers two main evolutionary parameters: *ν*, which is the ratio of the current effective population size (*N*_Curr_) to the ancestral effective population size (*N*_Anc_), and *τ*, the time since the most recent instantaneous size change in units of generations scaled by *2N*_Anc_ (Gutenkunst et al. 2009). For each species, we found the maximum-likelihood estimates (MLEs) of *ν* and *τ* by computing the expected SFS under a given set of parameters and evaluating its fit to the observed SFS (See Methods, Fig. 2c and d, supplementary fig. S3, Supplementary Material online).

As an example, we highlight the demographic inference of two species, *Bacteroides xylanisolvens* (Fig. 2a) and *Ruminococcus bromii* (Fig. 2b). For both species, a two-epoch demographic model best fits the data (Fig. 2e and f). Analysis of the SFS of *Bacteroides xylanisolvens* reveals an effective population size contraction in the range of 0.02 to 0.66 times the ancestral effective population size on a time-scale of roughly ∼58 to ∼9,000 years ago (these values represent the respective 95% confidence intervals of ν as well as τ rescaled to years, (supplementary fig. S4, Supplementary Material online) (See Methods). By contrast, *R. bromii* experienced a population expansion between 3.57 to 3.99 times the ancestral effective population size on a time-scale of roughly ∼44,000 to 63,000 years ago. Confirming these inferences, qualitatively, the observed SFS of *Bacteroides xylanisolvens* has an excess of common variants compared to a one-epoch model, consistent with a contraction, whereas *R. bromii* has an excess of rare variants, consistent with an expansion in effective population size.

Across the 39 species for which we were able to infer demographic models, we found that the two-epoch demographic model best fits the data in all analyzed bacteria except for *Bacteroides stercoris*, *Bacteroides plebeius*, and *Roseburia intestinalis*, all of which were instead better fit to a three-epoch model (supplementary table S3, Supplementary Material online). Across all the species best-fit to a two-epoch demographic model, ν ranges from ∼0.0685 to ∼5,933, indicating that some bacterial species have experienced a contraction (*n* = 10) while others have experienced an expansion (*n* = 26). In the three cases where *ν* is extremely large (>100 for the species *Dialister invisus, Alistipes shahii, and Bacteroides vulgatus*), the likelihood surface is very flat and confidence intervals exceptionally large, indicating substantial uncertainty in such large estimates of *ν* for these species (supplementary fig. S4, Supplementary Material online and supplementary table S4, Supplementary Material online). Finally, when converted to years (See Methods), MLEs for the timing of population size changes approximately fall between 5.0 × 10^2^ to 8.6 × 10^5^ years ago (supplementary table S4, Supplementary Material online). This range of timescales span many important periods of human history, including industrialization (10^2^ years ago), the transition to agriculture (10^4^ years ago), and out of Africa migrations (10^4^ to 10^5^ years ago).

Together, these findings suggest that commensal gut microbial populations have complex and diverse demographic histories, with some species experiencing substantial population size changes and others exhibiting more stable trends. Additionally, the approximate historical timescales of inferred demographic changes overlap with key events in human history, raising several interesting questions about how human behaviors may have selected for or against certain gut commensal microbiota, though this is an avenue for future work (See Discussion).

### DFEs of gut Commensal Microbiota Display Phylogenetic Trends

**Fig. 3.**
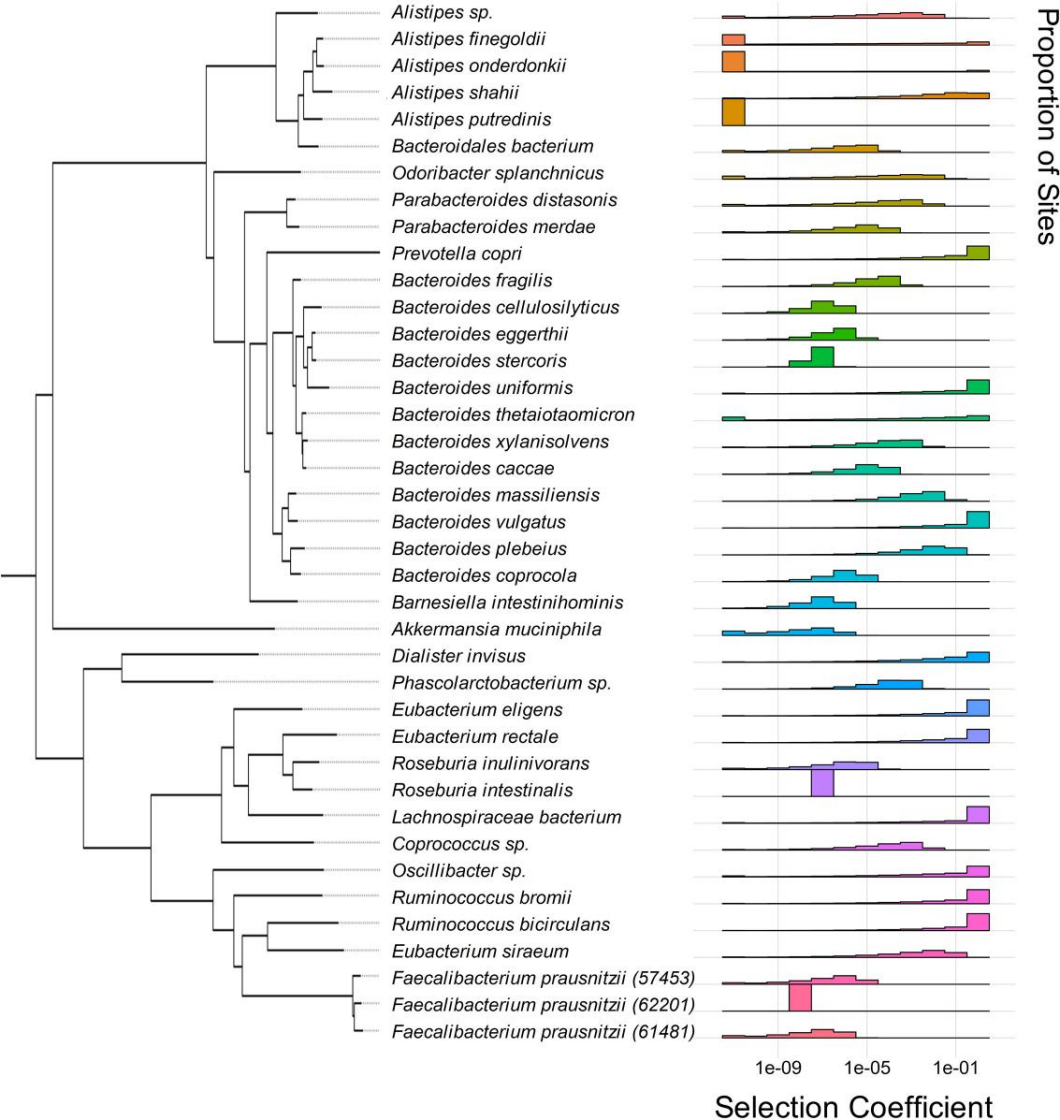
Phylogenetically sorted distributions of fitness effects. DFEs were estimated for each species assuming a gamma distribution. For each species, the *x*-axis denotes the log_10_ scaled discrete bin of selective effect, while the *y*-axis denotes the proportion of nonsynonymous mutations which fall into each bin.

We next turned our attention to inferring the fitness effects of nonsynonymous mutations. As seen in supplementary fig. S2a and b, Supplementary Material online, the observed SFS of synonymous SNPs differs substantially from that of nonsynonymous SNPs, with there being a higher proportion of singletons found among nonsynonymous SNPs compared to putatively neutral synonymous SNPs. This difference suggests the presence of strong purifying selection acting on nonsynonymous mutations (Bustamante et al. 2001; Williamson et al. 2005; Eyre-Walker et al. 2006; Eyre-Walker and Keightley 2007; Boyko et al. 2008) consistent with other bacterial populations (Hughes 2005; Cornejo et al. 2013). As such, we inferred the DFEs of nonsynonymous mutations in the 39 common commensal bacterial species for which we inferred demographic models (Fig. 3).

To infer the DFE, we constructed SFSs for nonsynonymous SNPs from the same species for which we constructed SFSs composed of synonymous SNPs. We then used Fit∂a∂i (Kim et al. 2017) to infer the DFE. Since demographic history can affect the shape or skew of the SFS, Fit∂a∂i infers selection conditional on the demographic history that was inferred from the synonymous SFS.

We examined the fit of several functional forms of the DFE to the SFS (Kim et al. 2017). One such functional form is a gamma distribution, characterized by *shape* and *scale* parameters that generate flexible distributions. Another such functional form is the neu + gamma distribution, which is a mixture distribution of a gamma distribution and a point-mass on neutrality. The neu + gamma distribution has one additional parameter compared to the gamma distribution, describing the proportion of neutral mutations. The model parameters that produce a nonsynonymous SFS most closely resembling the observed nonsynonymous SFS are considered representative of the underlying evolutionary parameters. We compared the AICs of the gamma-distributed DFE (Fig. 3) with the neu + gamma-distributed DFE (supplementary fig. S5, Supplementary Material online), and found that there was no significant improvement in fit for the neu + gamma compared to the gamma, except in the case of a single species, *Prevotella copri* (supplementary table S5, Supplementary Material online). Given a general absence of improved model fit for the neu + gamma model over the gamma model, below we further analyze the gamma-distributed DFEs.

There is considerable variation in the degree of deleterious selection coefficients (*s*) expected for each of the species analyzed. For example, in *R. bromii*, we inferred that 7.0% of nonsynonymous mutations were weakly deleterious or neutral (|*s*| < 10^−6^), 21.4% were moderately deleterious (10^−6^ < |*s*| < 10^−2^), 22.5% were highly deleterious (10^−2^ < |*s*| < 0.5), and 49% were lethal (|*s*| ≥ 0.5). By contrast, in *B. fragilis*, we inferred that 18.4% of mutations were weakly deleterious or neutral (|*s*| < 10^−6^), 81.6% were moderately deleterious (10^−6^ < |*s*| < 10^−2^), and no mutations were found to be highly deleterious (10^−2^ < |*s*| < 0.5) or lethal (*s*| ≥ 0.5).

We phylogenetically ordered the 39 inferred gamma-distributed DFEs (Fig. 3) and neu + gamma-distributed DFEs (See Methods; supplementary fig. S5, Supplementary Material online). This visualization revealed some evidence of phylogenetic trends in the DFE of the gut microbiome. For example, both species in the *Ruminococcus* genus were inferred to have DFEs skewed toward highly deleterious and lethal mutations (on average, ∼76.4% of new mutations are inferred to have |*s*| > 10^−2^). By contrast, many of the species in the *Bacteroides* genus have DFEs skewed toward weakly or moderately deleterious mutations (on average, ∼82.5% of sites are inferred with |*s*| < 10^−2^).

To quantify whether gamma-distributed DFEs between all pairs of the 39 species in our dataset are distinct versus the same, we implemented a likelihood ratio test (LRT) similar to that in Huber et al. (2017) (See Methods). Specifically, we compared the log-likelihood of two models: (1) a full model where pairs of species have independently inferred *shape* and *scale* parameters and (2) a constrained (null) model in which pairs of species have the same *shape* and *scale* parameters.

Using this framework, we found that the DFE is significantly different in 334 out of 741 (∼45%) pairs of species, after Bonferroni correction. Comparisons of DFEs between pairs of species belonging to the same genera versus different genera reveal some evidence of phylogenetic congruence of the DFE among closely related species. Concretely, of 334 significantly different DFEs, 284 (∼84%) of such tests were between species of different genera, while the remaining 50 (∼16%) were between species within the same genera, suggesting that differences in the DFE often arise between species that are more distantly related.

Supporting the observation that DFEs may be more different among distantly related species, we found that LRT statistics for between-genera comparisons are significantly elevated compared to within-genera comparisons (*P* = 0.008, two-sided Wilcoxon rank-sum test, supplementary fig. S6, Supplementary Material online). After permuting species labels (See Methods), there is no longer a significant difference. We also found that the difference in mean selection coefficient (E[*s*]) is significantly higher for between-genera comparisons versus within-genera comparisons, suggesting that *s* truly differs rather than there being a difference in statistical power (supplementary fig. S6, Supplementary Material online).

To more formally test whether the DFE parameters covary with phylogeny following Brownian motion, we estimated Pagel's *λ* (See Methods). We considered four parameters related to the DFE: the proportion of neutral mutations (i.e. |*s*| < 10^−6^), the mean selection coefficient (E[*s*]), the ancestral effective population size (*N*_Anc_), and the *shape* parameter of the best-fit gamma-distributed DFE. These analyses yielded some evidence of phylogenetic signal for the *shape* parameter (Pagel's *λ* ∼ 1.032, *P* = 4.9 × 10^−10^); however, this phylogenetic signal is supported by only two outlier species: *F. prausnitzii (62201)*, and *R. intestinalis*. Removing these two species from our dataset resulted in no evidence of phylogenetic signal (Pagel's *λ* = 7.17 × 10^−5^, *P* = 1.0). Thus, while there are greater differences in the DFE between species more distantly related compared to those more closely related, more generally, the DFE does not seem to have evolved via Brownian motion across the entire phylogeny.

We also considered if there are phylogenetic trends for DFEs when the selection coefficient, *s*, is scaled by *2N*_Anc_ (supplementary fig. S7a, Supplementary Material online) (Huber et al. 2017; Kim et al. 2017; Huang et al. 2021). *2N*_Anc_*s* provides insight into how selection and drift act together, whereas *s* provides insight into the relative fitness of individual mutations. Thus, we next compared whether the distribution of *2N*_Anc_*s* differs across species (supplementary fig. S7b, Supplementary Material online). We found that the LRT comparing pairs of DFEs is significant in 458 instances (∼62%), after Bonferroni correction (supplementary fig. S7b, Supplementary Material online). Of these 458 significantly different DFEs, 408 (∼89%) were between species of different genera, consistent with the results above performed with *s* showing that DFEs are more similar among closely related species. However, similar to our analyses of DFEs parameterized with *s*, Pagel's *λ* yields no evidence that the population-scale DFE covaries with phylogeny.

### Demographic and DFE Inference is Robust to the Removal of Low-Recombination Regions and Selective Sweeps

One of the main assumptions of SFS-based analyses is free recombination between sites. Given that rates of recombination can vary across the genome, we asked whether excluding regions of low recombination would result in different demographic and DFE inferences. To do so, we leveraged recent estimates of recombination rates (Liu and Good 2024) inferred from the same datasets analyzed in our study. For 18 species for which there were available recombination maps, we removed sites from the SFS found in genomic regions with recombination rates less than the inferred genome-wide median rate (See Methods).

Unaccounted-for selective sweeps may also confound inferences made from the SFS, as linked selection can result in skews in the SFS (Braverman et al. 1995; Fay and Wu 2000). In the 18 species for which we controlled for recombination, we excluded sites inferred to be undergoing a selective sweep (Wolff and Garud 2023), as well as 1,000 base pairs flanking the selective sweeps. These sweeps were inferred from the same datasets analyzed in this study. After removing low-recombination regions and selective sweeps, on average, the “filtered SFSs” retained ∼47.2% of SNPs compared to the “full SFS”.

**Fig. 4.**
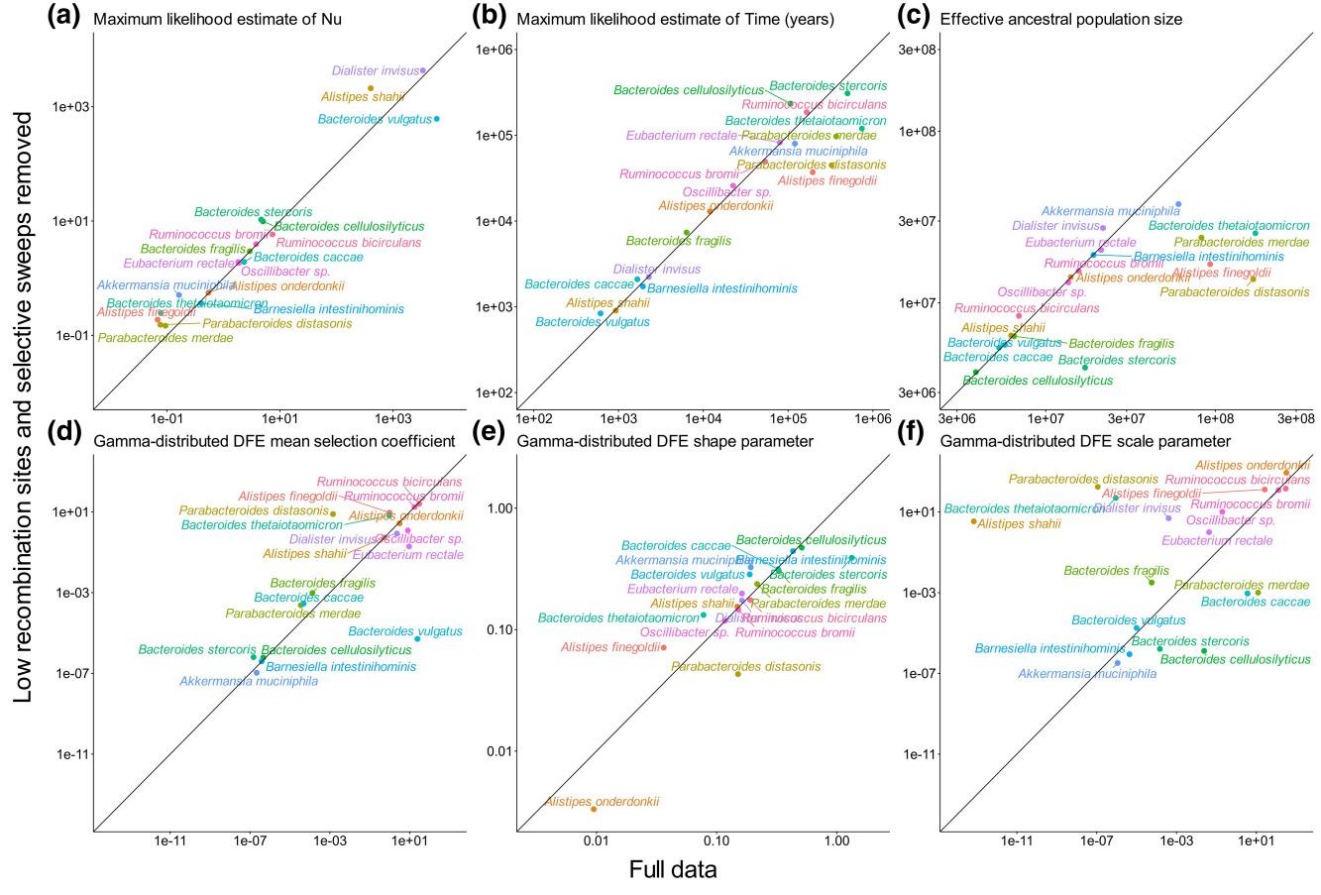
Comparison of evolutionary parameters inferred from the full SFS (*x*-axis) versus after low recombination and selective sweeps are removed (filtered SFS) (*y*-axis). The solid black-line represents the *y*  *=*  *x* trend for each panel. a) MLE of *ν*; b) MLE of time; c) MLE of the ancestral effective population size; d) MLE of the mean selection coefficient of Gamma-distributed DFE; e) MLE of the *Shape* parameter of Gamma-distributed DFE; f) MLE of the *Scale* parameter of Gamma-distributed DFE.

Figure 4 shows the comparison of demographic and DFE parameters inferred with the full versus filtered SFSs (supplementary tables S8 and S9, Supplementary Material online). The parameters *ν*, *τ*, E[*s*], *shape*, and *scale* inferred from both SFSs are correlated (*R*^2^ = 0.27, 0.27, 0.41, 0.43, 0.60, respectively); however, one parameter, N_Anc_, has a lower *R*^2^ of 0.20. This lower *R*^2^ is largely driven by the ∼5 species with the largest *N*_Anc_, whereby *N*_Anc_ in the filtered SFS is inferred to be smaller than in the full SFS. The reduction in *N*_Anc_ among these species could be due to recombination acting more efficiently in larger populations. In recombining bacterial populations, diversity may be reduced in regions where recombined fragments are transferred from one genome to another (Liu and Good 2024). Understanding the full impact of recombination on SFS inferences in greater detail is an avenue for future research. Despite this, the high correlation of inferred parameters with and without control for low recombination and selective sweeps suggests that demography and DFE inferences are largely robust to violations of independence in the SFS.

### Differences in the DFE Between Core Versus Accessory Genes are Largely Driven by Drift

**Fig. 5.**
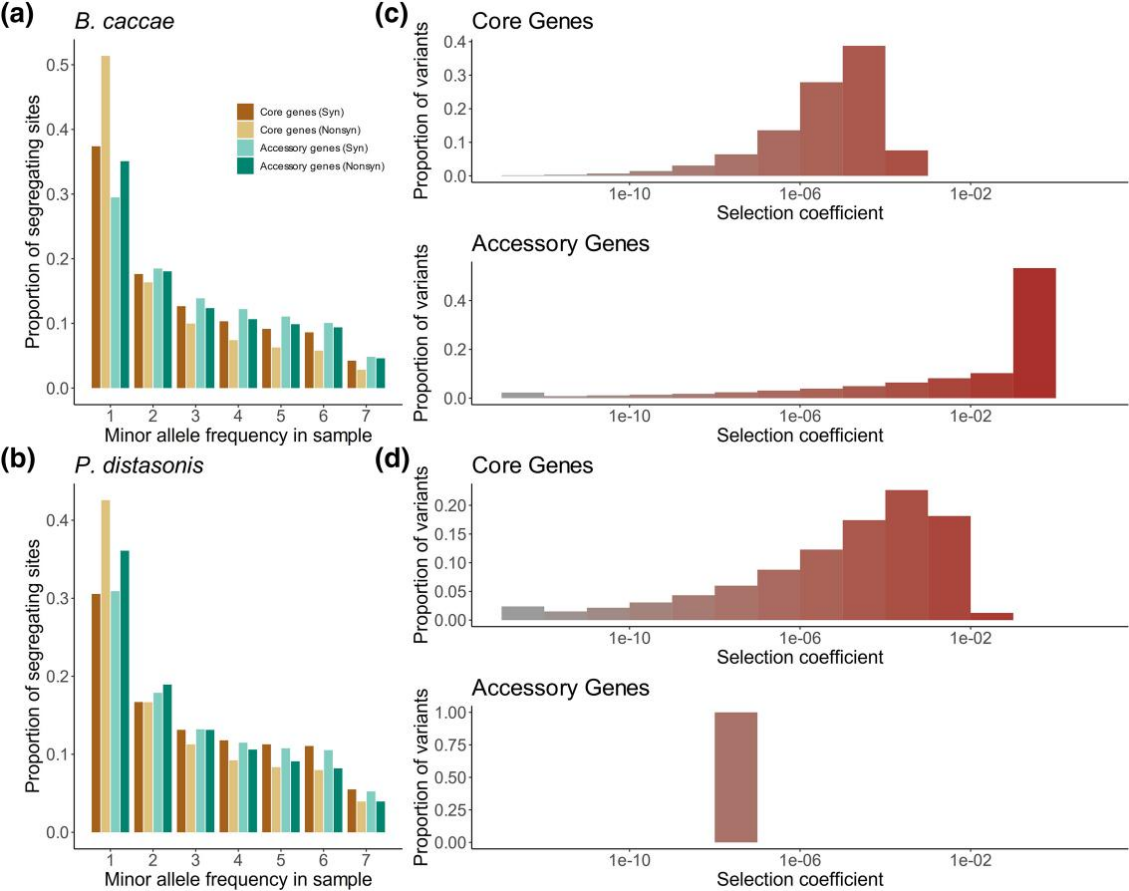
Differences in selective effects between core and accessory genes. Empirical site-frequency spectra of synonymous and nonsynonymous SNPs from core genes and accessory genes for two species: a) *Bacteroides caccae* and b) *Parabacteroides distasonis*. Gamma-distributed selection coefficients (*s*) of new mutations are shown for c) *Bacteroides caccae* and d) *Parabacteroides distasonis*. For each species, the *x*-axis denotes the log_10_ scaled discrete bin of selective effect, while the *y*-axis denotes the proportion of mutations which fall into each bin. Bins are color coded from gray to dark red according to the inferred lethality on the *x*-axis. See supplementary fig. S10, Supplementary Material online for similar plots for all species analyzed.

Thus far, our analyses only have considered core genes present in ≥95% of samples. Next, we evaluated whether accessory genes present in 30% to 70% of samples have different demographic histories or DFEs from core genes. To ensure that at least 14 genomes could be sampled even for the most rare genes considered (30% prevalence), we analyzed the 18 out of 39 species with (1) at least 47 QP samples (Fig. 1a) and (2) with models of demography and selection inferred from the accessory genome that adequately fit the SFS (Fig. 5c, supplementary fig. S7, Supplementary Material online).

By visual inspection, the synonymous SFSs constructed from the accessory genes typically exhibit a proportional depletion of rare variants relative to the SFSs constructed from core genes of the same species (Fig. 5a and b and supplementary fig. S8, Supplementary Material online). This signature is consistent with possible contractions in effective population size due to demography and/or reduced linked selection.

We first investigated if accessory versus core genes have distinct demographic histories. For 17 of the 18 species analyzed, we found that two-epoch demographic models best fit the data (supplementary table S6, Supplementary Material online). Moreover, we found that on average, ν was depressed for accessory genes compared to core genes, suggesting stronger population contractions and/or weaker population expansions in the accessory genome relative to the core genome (supplementary table S6, Supplementary Material online, supplementary fig. S9, Supplementary Material online). Additionally, for four of the species, *N*_Anc_ was found to be higher for accessory genes, while for nine of the species, *N*_Anc_ was found to be higher for core genes, suggesting overall differences in effective population sizes, and differing impacts of drift on core versus accessory genes.

Next we inferred the DFE for accessory genes (supplementary table S7, Supplementary Material online). Like core genes, accessory genes frequently harbor an excess of rare nonsynonymous variants compared to rare synonymous variants. However, core genes have a greater excess of rare nonsynonymous variants compared to rare synonymous variants suggesting that the DFEs of core genes may be more deleterious (Fig. 5a and b and supplementary fig. S8, Supplementary Material online). To test for a systematic difference in the DFE between core and accessory genes, we performed a LRT (See Methods) of similarity of DFEs inferred from accessory and core genes at two levels of selection: individual selective effects (*s*) and population-scaled selective effects (*2N*_Anc_*s*). We found that the DFE parameterized by *s* was significantly different in six species, with core genes being more deleterious in four species, and accessory genes being more deleterious in two species. All of these six species also had an even greater significant difference in *2N*_Anc_*s*. Specifically, when comparing the DFE between accessory and core genes with *2N*_Anc_*s*, we found that 10 species had a statistically significant difference in *2N*_Anc_*s* between accessory and core genes with six species having a more deleterious *2N*_Anc_*s* in the core genome and four species having a more deleterious *2N*_Anc_*s* in the accessory genome.

For the six cases where *2N*_Anc_*s* was higher in the core genome, *N*_Anc_ for the core genome was also inferred to be greater than or equal to *N*_Anc_ for the accessory genome. Similarly, for the four cases where *2N*_Anc_*s* was higher in the accessory genome, *N*_Anc_ for the accessory genome was also inferred to be greater than or equal for the core genome. This suggests that *N*_Anc_ may be driving differences in the DFE at the resolution of population-scale selection; however, differences in selection also arise at the level of individual mutations (*s*), suggesting that while drift is likely a driving force for differences in the DFE between core and accessory genes, selection still plays an important role.

## Discussion

In this study, we infer the population demographic histories and DFEs of new mutations of almost 40 prevalent human commensal gut bacteria. To do so, we used a SFS-based approach that is commonly used in eukaryotes (Gutenkunst et al. 2009; Beichman et al. 2018) but has been rarely used for bacterial populations (Cornejo et al. 2013; Pepperell et al. 2013; Mortimer et al. 2017). We found that commensal microbiota have experienced a range of effective population size changes over the course of human history. Additionally, we inferred finer distributions of fitness effects compared to previous work, which estimated the proportion of sites experiencing a single selection coefficient rather than a range of coefficients (Cornejo et al. 2013; Garud et al. 2019). Finally, we found that closely related species exhibit more similar DFEs than those from different genera, and that differences in the DFE between core versus accessory genes may largely be driven by differences in effective population size.

In our inferences, we assume that the SFS for synonymous SNPs accurately reflects demographic history. However, a myriad of evolutionary processes can affect the SFS, and thus have potential confounding effects (O’Fallon 2010; Lapierre et al. 2017). One such process is purifying selection acting on synonymous mutations, which can reduce diversity and skew the SFS toward rare variants (Chen et al. 2017; Johri et al. 2021; Murphy et al. 2022), potentially giving rise to false inferences of expansion. However, recently we found via a simulation study (Martinez I Zurita et al. 2024) that only extreme scenarios of strong negative selection (*s* = −0.001) acting on all synonymous mutations results in false inferences of severe (>100×) expansions. When fewer synonymous mutations are subjected to selection, the falsely-inferred expansions are smaller in magnitude than most of the expansions inferred in our study. Further, the fact that 10 out of 39 species we analyzed underwent population contractions suggests widespread selection on synonymous mutations is not driving the demographic signals we uncover.

Another evolutionary force that can create confounding signatures is the presence of selective sweeps, which can also generate an excess of rare variants (Braverman et al. 1995; Fay and Wu 2000). Additionally, while recombination seems to be common and frequent in human gut microbiota, local dips in recombination rates along the genome could result in linked effects between neutral and non-neutral sites. To our knowledge, here we perform the first systematic empirical investigation across multiple species on the impact of recombination and selective sweeps on demographic inference. The finding that demographic histories and DFEs are robust to the presence of selective sweeps as well as inclusion of heterogeneous recombining regions is important beyond our study, as SFS-based analyses are frequently employed across the tree of life without full knowledge of potential confounders.

Finally, false population contractions may be inferred if there is some force that inflates intermediate frequency synonymous variants, such as balancing selection or cryptic population substructure (Voight and Pritchard 2005; Lapierre et al. 2017). Balancing selection has been previously inferred using Tajima's *D* to act on a very small number of genes but not genome-wide (Moeller 2021). Thus, it is highly unlikely that the signals observed here for population contractions stem from balancing selection as we use genome-wide polymorphism data. While we controlled for population substructure (supplementary fig. S1, Supplementary Material online, Methods), both by removing closely related lineages and also by analyzing clades that display evidence of recombination among member lineages, we acknowledge there still could be residual cryptic population structure confounding analyses. Future work, potentially leveraging orthogonal signals like haplotype sharing, may be useful for uncovering different features of the demographic history and population structure that our current approach misses.

Despite their simplicity, basic aspects of our inferred demographic models concur with previous estimates of *N*_Curr_ made in the literature (supplementary fig. S11, Supplementary Material online). We found that MLEs for *N*_Curr_ typically fall between 10^6^ to 10^10^, in agreement with a previous study that estimated *N*_Curr_ using dN/dS to be in the range of 10^6^ to 10^9^ for >150 commensal, pathogenic, and free-living bacterial species (Bobay and Ochman 2018). Three of the species that we analyzed have *N*_Curr_ on the order of >10^9^, but the likelihood surfaces for these species are extremely flat, yielding almost equivalent log likelihoods for a wide range of values as low as 10^8^ (supplementary table S4, Supplementary Material online, supplementary fig. S3, Supplementary Material online).

It is striking that many of the demographic changes we infer coincide with key events in human history, such as the industrial revolution, the transition to agriculture, and human migration out of Africa. Even with a lower assumed mutation rate of 10^−10^ per base pair per generation (Barrick and Lenski 2013) (See Methods), our estimate of time in years still would span these significant human epochs. It is important to note that when setting up our analyses, we did not specify any particular time frame for the events to occur—rather, that the majority of demographic events occurred between 10^3^ to 10^6^ years ago is the outcome of our inferences. Specifically, we searched the parameter space of demographic events ranging from the present day to a maximum of 0.15 × *2N*_Anc_ generations ago (corresponding to a maximum of ∼7.5 million years ago on average), and could have inferred population size change events at any point in that time frame.

It is tempting to speculate that events like the agricultural revolution may have been causal of changes in effective population sizes among gut commensals. For example, Cornejo et al. inferred a population expansion for the cavity-causing pathogen *Streptococcus mutans* ∼10,000 to 20,000 years ago, potentially due to new available ecological niches opening up as a consequence of changes in diet during the agricultural revolution (2013). While the direction of inferred population size changes concur with changes in relative abundances for some species in industrialized versus traditional populations (e.g. we infer expansions for eight of ten *Bacteroides* species that have been previously shown to be higher in abundance in industrialized populations due to dietary shifts (Sonnenburg and Sonnenburg 2019)), this is not universally true. For example, we inferred a population expansion for *Prevotella copri* despite it being at markedly lower prevalence and abundance in industrialized versus traditional populations due to reductions in plant-based carbohydrates in western diets (Wu et al. 2011; Sonnenburg et al. 2016; Tett et al. 2019). It is possible that more sophisticated models can provide more biological insight into the population size changes experienced by gut microbiota as our models are necessarily simple and likely do not reflect the full set of demographic changes that have transpired for each species. The resolution of our ability to distinguish more sophisticated models is limited, however, by our sample size. For example, our investigation into two-epoch versus three-epoch models generally resulted in very similar AICs and LLs, making it difficult to distinguish between the simple models we inferred versus more complex models (supplementary fig. S2, Supplementary Material online). One-epoch models (models lacking any population size change), however, tended to have significantly worse AICs and thus were easier to reject. Future research leveraging larger sample sizes and possibly also time series data will be needed to infer more sophisticated models for human gut commensals and to make more explicit connections to human history and human health.

Beyond inferring a range of demographic histories across species, we also found differences in their DFEs. Interestingly, we found that phylogenetically related species have more similar DFEs than distantly related species (supplementary fig. S6, Supplementary Material online, Methods) potentially implying functional similarities among closely related species. We note that both differences in *s* and *N*_Anc_ could be driving differences in the DFE across species, though we find a greater number of differences between pairs of species when comparing the DFE in terms of *2N*_Anc_*s* (458 pairs) rather than *s* (334 pairs), suggesting that variation in ancestral population size plays a major role in driving differences in the DFE across species. Similarly, the differences in the DFEs for core versus accessory genes are less likely due to differences in *s* and instead largely due to differences in *N*_Anc_.

Finally, it is worth noting that our DFE and demographic history inferences rely on genotypes supported by a majority of reads within hosts (See Methods), mitigating the impact of sequencing errors on our inferences. Consequently, our inferred DFEs represent what could be considered an “average across hosts and may not fully capture within-host DFE variations, which can potentially differ among individuals and environments (Dapa et al. 2023). Future research utilizing experimental evolution techniques (Shiver et al. 2023; Wong and Good 2024) will be pivotal in understanding differences between within-host and across-host DFEs.

In summary, this work advances our understanding of the evolutionary dynamics of human gut microbiota, highlighting the influence of demographic history and selective forces on genetic variation. Our findings underscore the complexity of these interactions and their implications for the human microbiome, which future work will undoubtedly further elucidate.

## Methods

### Data

Raw whole-genome shotgun sequencing reads for North American metagenomic samples analyzed in this study were downloaded from the Human Microbiome Project Consortium (Human Microbiome Consortium 2012; Lloyd-Price et al. 2017). Accession numbers associated with this data are in supplementary table S1, Supplementary Material online.

This dataset included 471 fecal samples from 250 healthy individuals. A subset of these 250 individuals were sampled at two or three time points. We analyzed data from a single time point per host per species in the North American dataset. Additionally, since previous work has shown that technical and sample replicates of the same fecal sample have little genomic variability (Nayfach et al. 2016; Maghini et al. 2024), we merged FASTA files for technical and sample replicates from the same time point prior to calling species, SNPs and CNVs to increase coverage as in Garud et al. (2019).

For European gut microbiomes, there were 250 fecal samples available from 125 pairs of human adult twins (Xie et al. 2016). We analyzed data from a single randomly selected twin to better approximate a random sampling of the population. Additionally, we analyzed one family member chosen at random from Korpela et al. (2018).

For Chinese gut microbiomes (Qin et al. 2010), we analyzed 185 fecal samples from 185 healthy individuals.

Relevant data, including accession numbers are in supplementary table S1, Supplementary Material online.

### Identification of Bacterial Species, Genes, and SNPs

We used a standard reference-based approach called MIDAS to quantify bacterial species abundances and gene and SNP content (Nayfach et al. 2016).

The first step of the MIDAS pipeline is to determine the species present in each set of sample(s) for each host. MIDAS quantifies the relative abundance of species in a given sample by mapping sequencing reads to a reference database of single-copy “marker” genes unique to each species. We used database version 1.2 (Nayfach et al. 2016), downloaded on November 21st, 2016. A species was considered present if it had an average marker gene coverage of at least 3 × for a given sample.

Second, MIDAS quantifies SNPs for each species for each sample. Similar to the “species” step, this “SNP” step leverages a standard reference-based approach in which reads are mapped to a single genome per species. To avoid reads mapping spuriously to species not present in the dataset, reads were mapped only to species truly present in the sample as per the species step defined above. Specifically, the reference panel consisted of the union of species found in a host at any time point, as per Garud et al. (2019). Mapping was done with Bowtie2 (Langmead and Salzberg 2012) with the following default MIDAS mapping thresholds: global alignment, MAPID ≥ 94.0%, READQ ≥ 20, ALN_COV ≥ 0.75, and MAP ≥ 20.

MIDAS annotates SNPs as either 1D or 4D, indicating codon degeneracy. For example, 1D means that any nucleotide change will result in an amino acid change, resulting in a nonsynonymous site. By contrast, 4D means that any nucleotide change will result in a synonymous site. When constructing synonymous SFSs, only 4D sites were considered, and when constructing nonsynonymous SFSs, only 1D sites were considered.

Finally, MIDAS identifies gene CNVs by aligning reads to a pangenome, i.e. the union of all genes found in any strain present in the MIDAS database for a given species. Once again, reads were mapped to pangenomes only for those species considered to be present. Standard MIDAS read coverage parameters were used for calling CNVs and as described in Garud et al. (2019).

Since there may be orthologs of the same gene present in multiple species, these genes can potentially result in spurious read donating and recruiting. Therefore, any gene with ≥95% ANI between different species' pangenomes was excluded from further analysis. Genes with ≥95% ANI with another gene in multiple species' pangenomes were previously identified in Garud et al. (2019).

To identify which genes to include in our analysis, we considered the copy number of each gene. Genes were included in our data set if their estimated copy number was ≥0.3 and ≤3.0 (Garud et al. 2019), to avoid including genes with insufficient read support and genes with abnormally elevated copy numbers, potentially due to spurious read mapping. Additionally, any gene with copy number values >3.0 in any host was excluded from the entire dataset to once again control against spurious read mapping. Next, to differentiate between core and accessory genes, we considered the prevalence of each gene: core genes were identified as genes with prevalence ≥95%, while accessory genes were identified as genes with prevalence between 30% and 70%.

Once core and accessory genes were identified, sites were included using a minimum sample depth of 20 × coverage, and a minimum site depth of 20 reads.

### Quasi-Phasing

To estimate quasi-phased haplotypes from individual hosts, we identified hosts in which the lineage structure of their bacteria was simple enough to assign alleles to the dominant lineage with high confidence as described in Garud et al. (2019). Since both our paper and Garud and Good et al. utilize the same data for analyses, we used the same sample × species identified in Garud and Good et al. that were classified as quasi-phaseable (2019). We then estimated the genotype of the dominant lineage by assigning the major allele of each site only if it had a frequency of 0.8 or greater. Any site with a major allele frequency between 0.5 and 0.8 was considered missing data.

### Phylogenetic Ordering

To order species phylogenetically (such as in Fig. 3), we used the phylogenetic tree from Nayfach et al. (2016). We abstracted a subtree of relevant species using the APE package (version 5.8) in R (Paradis and Schliep 2019).

### Calculating the SFS

We compute “folded” site-frequency spectra (SFS) for both synonymous and nonsynonymous sites from quasi-phased bacterial genomes. The folded SFS describes the distribution of minor allele frequencies across the genome. “Folded” indicates that since the ancestral versus derived state of alleles are unknown, any allele frequency (*f*) that is >50% becomes 1–*f*. Folded SFSs have been shown to yield accurate inferences of the DFEs of deleterious mutations (Keightley and Eyre-Walker 2007; Boyko et al. 2008; Kim et al. 2017).

The SFS can be represented as a vector, $X=[X_{1},X_{2},\ldots ,X_{n-1}],\;$ in which each element of the vector describes the number of SNPs at frequency *i* given *n* chromosomes. In other words, $X_{1}$ is the number of singletons, $X_{2}$ is the number of doubletons, etc. Thus, the SFS describes the number of QP samples a SNP appears in. A site is considered to be variable if at least one quasi-phased genome has a different allele from other quasi-phased genomes in our dataset.

### Projection of the SFS

The empirical SFS may show irregular peaks and valleys due to missing data (supplementary fig. S1b, Supplementary Material online), which may confound inference from and modeling of the SFS, as some sites are not called for all individuals. To diminish the confounding effects of missing data, we projected the empirical SFS down from a larger sample size to a smaller sample size of 14 haplotypes for all species. In other words, for all sites present in ≥14 hosts, we computed the expected frequency of each SNP in a sample of 14 haplotypes using a hypergeometric distribution (Gutenkunst et al. 2009). Sites with data in <14 hosts were omitted.

### Controlling for Recombination

A major assumption of SFS-based analyses is free recombination between sites. To control for low recombination regions, we inferred recombination maps using recent work from Liu and Good (2024). Liu and Good provide the first estimate to date of recombination rates across bacterial genomes for the 29 most prevalent and abundant species present in Westernized gut microbiomes using the same datasets analyzed in this paper (2024). To generate this recombination map, they inferred recombination transfer events between pairs of strains residing within the same host. To convert these transfer events to a rate of recombination per site, we computed the mean number of detected transfer events occurring in 1,000-bp windows across the core genome using any pair of strains with a transfer event in that region. We then removed sites from the SFS found in regions with a mean recombination rate less than the median recombination rate for each of the 18 species overlapping our dataset.

### Controlling for Selective Sweeps

Unaccounted-for selective sweeps and patterns of linked selection may confound inferences made from SFSs, as evolutionary signals from these forces may be incorrectly attributed to population demographic history. To control for selective sweeps, we masked regions of the genome that we previously inferred using the same dataset to have undergone selective sweeps (Wolff and Garud 2023). We also masked 1,000 bps flanking the selective sweeps.

### Inference of Demographic Models

We consider three model specifications: (1) a one-epoch model consisting of a constant effective population size, (2) a two-epoch model consisting of two epochs, i.e. two periods of time, separated by one effective population size change, and (3) a three-epoch model consisting of three epochs separated by two effective population size changes (Fig. 1c).

We use the statistical inference package, ∂a∂i (Gutenkunst et al. 2009) to infer two main demographic parameters for each effective population size change, *τ* and *ν*. The time of the population size change is represented by *τ*, in units of $\frac{generations}{2N_{Anc}\;}$, where *N*_Anc_ is the ancestral effective population size. The parameter *ν* denotes the ratio of effective population size after the population size change relative to *N*_Anc_.

To find the maximum-likelihood parameter estimates, we defined a grid of points over a likelihood surface parameterized by *τ* and *ν*. For a given point on the likelihood surface, ∂a∂i estimates the model spectrum expected under those evolutionary parameters using diffusion theory. The multinomial log-likelihood function is then used to assess the fit of the model spectrum to the empirical data as described in Gutenkunst et al. (2009). We then performed a gradient-descent based approach to evaluate points along the likelihood surface to find the MLEs. The pair of parameters which yielded the highest log-likelihood were assumed to represent the underlying evolutionary history. We did not force parameter bounds on the likelihood surface, allowing the gradient-descent search to evaluate the entirety of the log-likelihood surface. In addition, at least 25 initial parameter guesses, spanning a large range across the parameter surface, were evaluated to ensure that the likelihood surface did not converge at a local maximum specific to a set of starting parameters. We used the chi-square approximation to the log-likelihood ratio to obtain the critical values for the asymptotic 95% confidence intervals of the demographic parameters. The CIs included all parameter values within three log-likelihood units (df = 2, reflecting the two parameters) from the MLE.

### Estimation of Ancestral Population Size

We compute *N*_Anc_ from $\theta_{s}$ as:


(1) $$N_{Anc}=\frac{\theta_{s\;}}{4\mu L_{syn}}\;\;$$

where *μ* is the mutation rate per site per generation and $L_{syn\;}\;$ is the number of synonymous sites. $\theta_{s}$ is the population-scaled mutation rate, optimized as the scaling factor between the empirical data and the model spectrum that best fits the data. *μ* is assumed to be 4.08 × 10^−10^ per site per generation, based on experimental estimation for neutral evolution in bacteria (Drake 1991).

### Computation of Estimated Times of Demographic Size Changes

We convert the time parameter, *τ*, which is in units of $\frac{generations}{2N_{anc}\;}$, into units of time in years. We chose to assume a conservative generation time of one generation per day, based on estimates of bacterial growth (Gibbons and Kapsimalis 1967; Savageau 1983; Ghosh and Good 2022). Thus, we compute:


(2) $$Time\;(in\;years)\;=\frac{\tau 2N_{Anc}}{365}$$

### Inference of the DFEs of Nonsynonymous Mutations

To infer the distribution of fitness effects (DFE), we utilize the SFS of nonsynonymous variants. We use a statistical inference package, Fit∂a∂i (Kim et al. 2017). Fit∂a∂i uses a maximum-likelihood approach to infer the DFE from site-frequency spectra. We note that *s* in Fit∂a∂i refers to the change in relative fitness of the heterozygous genotype and is equivalent to the fitness in the haploid.

We examined two different probability distributions for the DFE: a gamma-distributed DFE, and a neutral + gamma-distributed DFE. Under a gamma-distributed DFE, the selective effect of a given mutation, denoted as *s*, is randomly drawn from a gamma distribution, parameterized by *shape*, denoted as *α* and *scale*, denoted as *β*. Under a neutral + gamma-distributed DFE, *s* is drawn from a gamma distribution, but a proportion of sites is assumed to be neutral, generating a point-mass at *s*  *=*  *0*, parameterized as *p_neu_*.

The parameters for the DFEs described above were inferred using Fit∂a∂i. Importantly, because demography can impact the SFS, we condition on the demographic models described above that we inferred from synonymous variants. Fit∂a∂i models the expected nonsynonymous SFS for a given combination of DFE parameters and a demographic history. Then, the fit to the data is computed using a Poisson log-likelihood function. In contrast with the multinomial likelihood, the Poisson likelihood additional requires an a priori assumption about the population-scaled mutation rate of nonsynonymous mutations, denoted as $\theta_{ns}$. $\theta_{ns}$ is obtained by multiplying the the best-fitting $\theta_{s}$ from demographic inference by the expected ratio of nonsynonymous to synonymous mutation rates. We assumed this ratio to be $\theta_{ns}/\theta_{s}=2.31$ (Huber et al. 2017). We found the DFE parameters that maximized the log-likelihood by gradient descent over a two-parameter likelihood surface parameterized by *α* and *β*. Similar to inference of demography, we did not force parameter bounds on the likelihood surface, allowing the gradient-descent search to evaluate the entirety of the log-likelihood surface. Furthermore, at least 25 initial parameter guesses, spanning a large range across the parameter surface, were evaluated to ensure that the likelihood surface did not converge at a local maximum specific to a set of starting parameters.

### Comparing DFEs Across Species

To quantify the similarities and differences between the DFEs of two species, we performed a LRT comparing the fit of models of the DFEs of nonsynonymous mutations assuming that the *shape* and *scale* parameters are the same in both species (the null model) versus a model where each species can have its own *shape* and *scale* parameters (the alternative model).

Consider two species, *A*, and *B*, for which we have independently inferred demographic histories, respectively denoted as $\Theta_{D,A}$ and $\Theta_{D,B}$. Conditional on these best-fitting demographic histories, we then independently fit the DFE for both species under a gamma-distributed model specification, yielding a *shape* parameter *α* and a *scale* parameter *β* for each species. To treat species *A* and *B* as independent (Boyko et al. 2008; Lawrie and Petrov 2014), we used a Poisson composite likelihood function identical to the likelihood function used in Huber et al., reproduced here (2017):


(3) $$\begin{array}{rl} & L(\alpha_{A},\;\beta_{A},\;\alpha_{B},\;\beta_{B}|\Theta_{D,A},\;\Theta_{D,B})\\ & =\overset{n-1}{\underset{i=1}{\prod }}\frac{E{\left(X_{i,A}|\alpha_{A},\;\beta_{A}.\;\Theta_{D,A},\;\theta_{A}\right)}^{X_{i,A}}}{X_{i,A}!}\;\cdot e^{-E(X_{i,A}|\alpha_{A},\beta_{A}.\;\Theta_{D,A},\theta_{A})}\cdot \;\\ & \;\overset{n-1}{\underset{i=1}{\prod }}\frac{E{\left(X_{i,B}|\alpha_{B},\;\beta_{B}.\;\Theta_{D,B},\;\theta_{B}\right)}^{X_{i,B}}}{X_{i,B}!}\;\cdot e^{-E(X_{i,B}|\alpha_{B},\beta_{B}.\;\Theta_{D,B},\theta_{B})} \end{array}$$

where *n* and *m* denote the sample size of species *A* and *B* respectively, and $X_{i,A}\;$ denotes the number of SNPs at frequency *i* for a given species, *A*.

To test whether the *shape* (*α*) and *scale* (*β*) parameters of species *A* differ from those in species B, we utilized the LRT similar to that proposed in Huber et al. (2017):


(4) $$\Lambda =\;\frac{L(\hat{\alpha_{A}},\;\hat{\alpha_{B}},\;\hat{\beta_{A}},\;\hat{\beta_{B}}\;|\;\hat{\Theta_{D,A}},\;\hat{\Theta_{D,B}}\;)}{L(\hat{\alpha_{A}=\alpha_{B},}\hat{\beta_{A}=\beta_{B}}|\;\hat{\Theta_{D,A}}\hat{,\;\;\Theta_{D,B}})}$$

The null hypothesis for this test is the likelihood of the constrained model, i.e. the case in which $\alpha_{A}=\alpha_{B}$ and $\beta_{A}=\beta_{B}$, while the full model allows for $\alpha_{A}\neq \alpha_{B}$ and $\beta_{A}\neq \beta_{B}$. We optimized the likelihood function under both the constrained model and the full model. We optimized the constrained model by considering a grid of values for *α* and *β*. We then used Fit∂a∂i to calculate a SFS of nonsynonymous variants for each pair of parameters for each species, and computed the log-likelihood for the fit of each model SFS to the data. Then, for each pair of parameter values, we summed the log-likelihood over both species. The pair of parameters with the highest log-likelihood was the MLE for this constrained null model.

Asymptotically, 2 *Λ* follows a $\chi^{2}$ distribution with two degrees of freedom, as the full model has two additional free parameters relative to the constrained model. The critical value for the LRT is approximately 6 for a critical region of 0.05 for a singular test; however, given that we perform 471 pairwise comparisons, we approximate the critical value as 18.30 to correct for multiple hypothesis testing.

### Comparing DFEs Between Core and Accessory Genes

Similar to the method by which we compare DFEs across species, we performed a LRT comparing DFEs of core versus accessory genes, whereby the null hypothesis is a constrained model which assumes that core and accessory genes have the same *α* and *β* parameters. The alternative hypothesis is a full model in which *α* and *β* may be different for core and accessory genes.

We perform comparisons of the DFE between core and accessory genes for eighteen species; thus, the critical value for this LRT is approximately 11.77, after Bonferroni correction.

### Phylogenetic Analyses of the DFE

To assess the phylogenetic signal of the DFE while controlling for the phylogenetic non-independence of species we considered four quantities: the proportion of neutral mutations (i.e. |*s*| < 10^−6^), the mean selection coefficient (E[*s*]), the ancestral effective population size (*N*_Anc_), and the *shape* parameter of the best-fit gamma-distributed DFE.

To assess the phylogenetic signal of these four quantities, we computed Pagel's Lambda (*λ*) using the R package “phylosignal” (Keck et al. 2016). This analysis was performed with `phylosignal` version 1.3.1.

## Supplementary Material

msaf010_Supplementary_Data

## Data Availability

All data analyzed in this paper is publicly available. Accessions for the data analyzed are available in supplementary table S1, Supplementary Material online.

## Code Availability

All code for the bioinformatics pipeline, downstream analysis, and figure generation are available at Github: https://github.com/garudlab/microbiome_demography_manuscript.
