# Supplementary material for: Inference of the Demographic Histories and Selective Effects of Human Gut Commensal Microbiota Over the Course of Human History
Source: Mol Biol Evol. 2025 Jan 22;42(2):msaf010. doi: 10.1093/molbev/msaf010 (PMC11824422; doi:10.1093/molbev/msaf010)
Supplement: msaf010_Supplementary_Data [file msaf010_supplementary_data.zip › mah2024demography_supplement.pdf]

SUPPLEMENT

Table of contents

Tables

Table S1: Fecal sample metadata	Pg 3
Table S2: Demographic inference for core genes	Pg 3
Table S3: Lower and upper bounds of 95% confidence intervals of ν and time for two-epoch demographic models	Pg 3
Table S4: DFE inference for core genes	Pg 3
Table S5: Demographic inference for accessory genes	Pg 3
Table S6: DFE inference for accessory genes	Pg 4
Table S7: LRT statistics for core vs. accessory genes	Pg 4
Table S8: Demographic inference for filtered SFS	Pg 4
Table S9: DFE inference for filtered SFS	Pg 4

Figures

Figure S1: Effects of control and downsampling on the site-frequency spectrum	Pg 5
Figure S2: Akaike information criterion for one-epoch, two-epoch, and three-epoch demographic models inferred in this paper	Pg 6
Figure S3: SFSs and likelihood surfaces for demographic and DFE inference with core genes	Pg 6
Figure S4: Confidence intervals for ν and τ for demographic inference from core genes	Pg 7
Figure S5: Neu+gamma-distributed DFE inferred from core genes	Pg 8
Figure S6: Statistics for comparing within-genera and between-genera DFEs	Pg 9-10
Figure S7: Likelihood ratio test comparing pairs of DFEs in 39 species	Pg 10

Figure S8: SFSs and likelihood surfaces for demographic inference from accessory genes	Pg 10
Figure S9: Empirical SFS of core and accessory genes	Pg 11
Figure S10: Summary statistics inferred from the core vs. accessory genome	Pg 11
Figure S11: Phylogenetically ordered analysis of core and accessory gene DFE	Pg 12
Figure S12: Distribution of N_{Curr} for core genes	Pg 13

Table S1: Fecal sample accession numbers

https://github.com/garudlab/microbiome_demography_manuscript/blob/main/Supplement/Supplemental_Table_1.tsv

We analyzed 693 healthy hosts from 4 datasets from North America (Human Microbiome Consortium 2012), Europe (Xie et al. 2016; Korpela et al. 2018), and China (Qin et al. 2010). Listed are the subject identifiers, sample identifiers, run accessions, country of the study performed, continent of the study performed, and visit number or twin identity (Human Microbiome Consortium 2012; Lloyd-Price et al. 2017).

Table S2: Demographic inference for core genes

https://github.com/garudlab/microbiome_demography_manuscript/blob/main/Supplement/Supplemental_Table_2.csv

Phylogenetically sorted summarizing table of demographic inference for core genes of 39 common commensal gut microbiota. Three model specifications were considered: a one-epoch demographic model, a two-epoch demographic model, and a three-epoch demographic model (Methods). Listed are the log likelihood, AIC, and maximum likelihood parameters for each species and model specification. τ is converted to time in years as described in the Methods. For all species except *Bacteroides stercoris*, *Bacteroides plebeius*, and *Roseburia intestinalis*, a two-epoch demographic model best fits the data.

Table S3: Lower and upper bounds of the 95% confidence intervals of ν and time for two-epoch demographic models

https://github.com/garudlab/microbiome_demography_manuscript/blob/main/Supplement/Supplemental_Table_3.csv

Phylogenetically sorted summarizing table of lower and upper bounds for ν and time in years since the most recent demographic event. Upper and lower bounds were computed using the 95% confidence interval of the log likelihood surface, approximated as the parameter space within 3 log likelihoods of the MLE (**Figure S1**) (Methods).

Table S4: DFE inference for core genes

https://github.com/garudlab/microbiome_demography_manuscript/blob/main/Supplement/Supplemental_Table_4.csv

Phylogenetically sorted summarizing table of DFE inference for core genes of 39 common commensal gut microbiota. Two model specifications were considered: a gamma-distributed DFE, and a neu+gamma-distributed DFE (Methods). Listed are the log likelihood, AIC, and maximum likelihood parameters for each species and model specification.

Table S5: Demographic inference for accessory genes

https://github.com/garudlab/microbiome_demography_manuscript/blob/main/Supplement/Supplemental_Table_5.csv

Phylogenetically sorted summarizing table of demographic inference for accessory genes of 18 common commensal gut microbiota. Listed are the log likelihood, AIC, and maximum likelihood parameters for each species and model specification. τ is converted to time in years as described in the methods. For ease of comparison, the ancestral effective population size inferred from core genes is shown as the right-most three columns.

Table S6: DFE inference for accessory genes
https://github.com/garudlab/microbiome_demography_manuscript/blob/main/Supplement/Supplemental_Table_6.csv
Phylogenetically sorted summarizing table of DFE inference for core genes of 18 common commensal gut microbiota. Listed are the log likelihood, AIC, and maximum likelihood parameters for each species and model specification.

Table S7: LRT statistics for core vs. accessory genes
https://github.com/garudlab/microbiome_demography_manuscript/blob/main/Supplement/Supplemental_Table_7.csv
Testing the null hypothesis that the DFEs for core and accessory genes from the same species follow the same gamma distribution. Two forms of null hypothesis were tested: one in which the selection coefficient s is assumed equal between core and accessory genes, and one in which the population-scaled selection coefficient, $2N_{\text{Anc}}s$ is assumed equal between core and accessory genes (Methods). Given 18 tests, the critical value for statistical significance after Bonferroni correction is approximately 11.77. *Alistipes putredinis* found no difference in the DFE between the core and accessory genes under either form of the null model.

Table S8: Demographic inference for filtered SFSs
https://github.com/garudlab/microbiome_demography_manuscript/blob/main/Supplement/Supplemental_Table_8.csv
Phylogenetically sorted summarizing table of demographic inference for filtered SFSs of 18 common commensal gut microbiota. Listed are the log likelihood, AIC, and maximum likelihood parameters for each species and model specification. τ is converted to time in years as described in the methods. For ease of comparison, the ancestral effective population size inferred from core genes is shown as the right-most three columns.

Table S9: DFE inference for filtered SFSs
https://github.com/garudlab/microbiome_demography_manuscript/blob/main/Supplement/Supplemental_Table_9.csv
Phylogenetically sorted summarizing table of DFE inference for filtered SFSs of 18 common commensal gut microbiota. Listed are the log likelihood, AIC, and maximum likelihood parameters for each species and model specification.

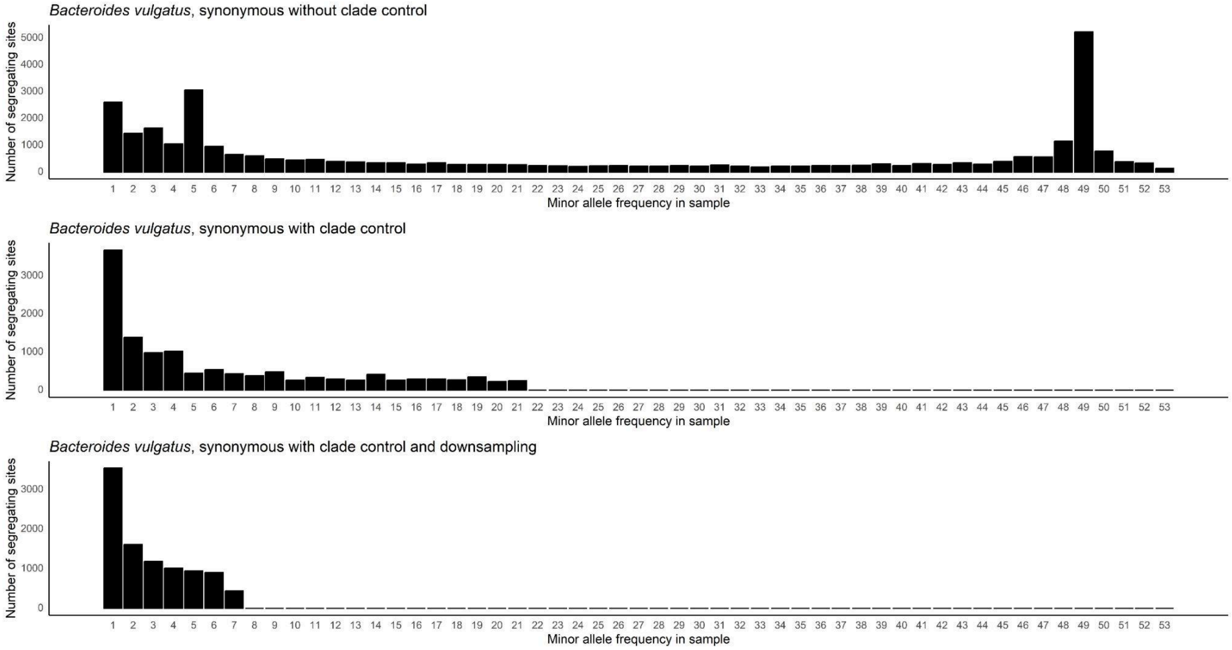

Figure S1: Effects of control and downsampling on the site-frequency spectra
https://github.com/garudlab/microbiome_demography_manuscript/blob/main/Supplement/Supplemental_Figure_1.jpg
Synonymous folded empirical site-frequency spectrum (SFS) of *Bacteroides vulgatus* when (**A**) including lineages from all clades, (**B**) when including lineages only from the largest clade and excluding any closely related lineages , and (**C**) when the samples from (**B**) are downsampled to 14 individuals . Each additional modification to the SFS produces a progressively smoother and more monotonic relationship amongst bins.

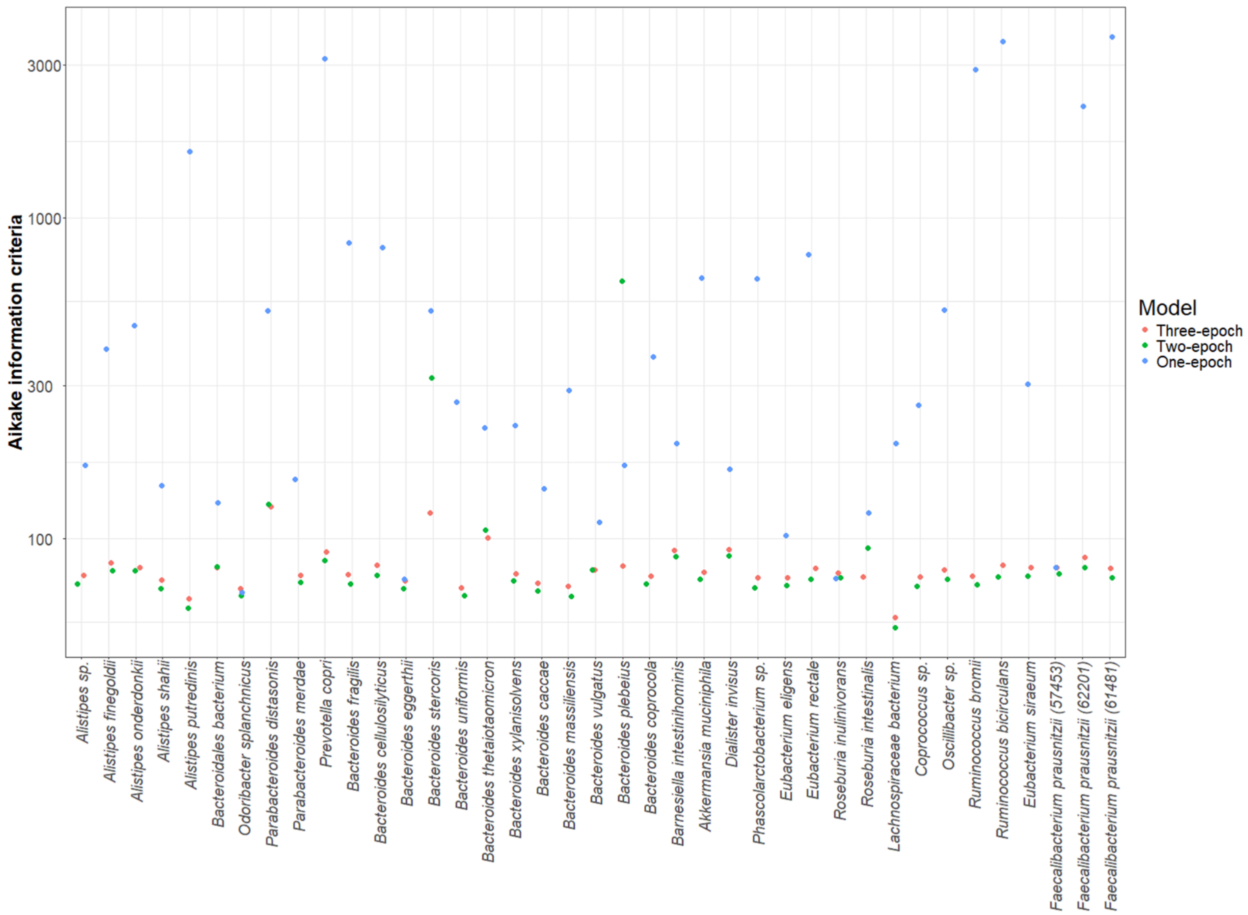

Figure S2: Akaike information criterion for one-epoch, two-epoch, and three-epoch demographic models inferred in this paper

https://github.com/garudlab/microbiome_demography_manuscript/blob/main/Supplement/Supplemental_Figure_2.jpg

Phylogenetically sorted panel of the Akaike information criteria computed for 1, 2, and 3-epoch models inferred in this paper for each species.

Figure S3: SFSs and likelihood surfaces for demographic and DFE inference with core genes (see attachment)

https://github.com/garudlab/microbiome_demography_manuscript/blob/main/Supplement/Supplemental_Figure_3.jpg

(Left) Site frequency spectra from the empirical data compared to those predicted from the maximum likelihood models for core genes from 39 common commensal gut microbiota. "MLE synonymous" shows the expected SFS produced by the ML demographic parameter estimates. "MLE nonsynonymous" shows the expected SFS produced by the ML demographic and selection parameters from a gamma-distributed DFE. (Right) 2-dimensional likelihood surfaces of population size, ν (in units of N_{Anc}) and time since the onset of the most recent demographic event, τ (in units of $\frac{generations}{2N_{Anc}}$). A dashed red line separates the parameter space between contractions and expansions. The maximum-likelihood demographic parameter estimates are

shown with an orange dot, and colored intervals denote the decrease in log-likelihood from the MLE. The light cyan regions (LL - 3) correspond to the asymptotic 95% confidence interval, from the chi-squared distribution with 2 degrees of freedom.

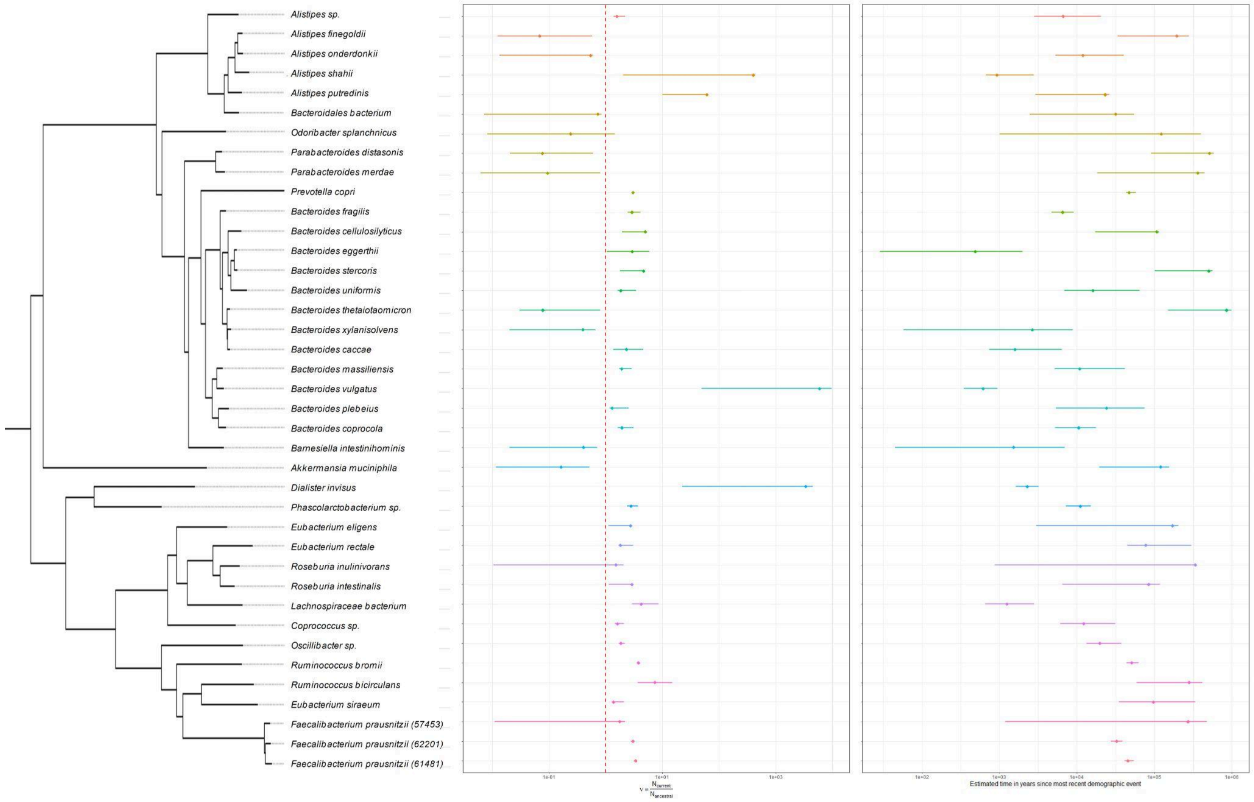

Figure S4: Confidence intervals for ν and τ for demographic inference from core genes
https://github.com/garudlab/microbiome_demography_manuscript/blob/main/Supplement/Supplemental_Figure_4.jpg
Phylogenetically sorted panels of 95% confidence intervals for ν (in units of N_{Anc}) (left) and time in years since the most recent demographic event (right). The maximum likelihood parameter estimate is indicated with a diamond. Confidence intervals are color coded by species using the same color scheme as **Figure 3**. For the left plot, a dashed red line separates the parameter space between contractions and expansions.

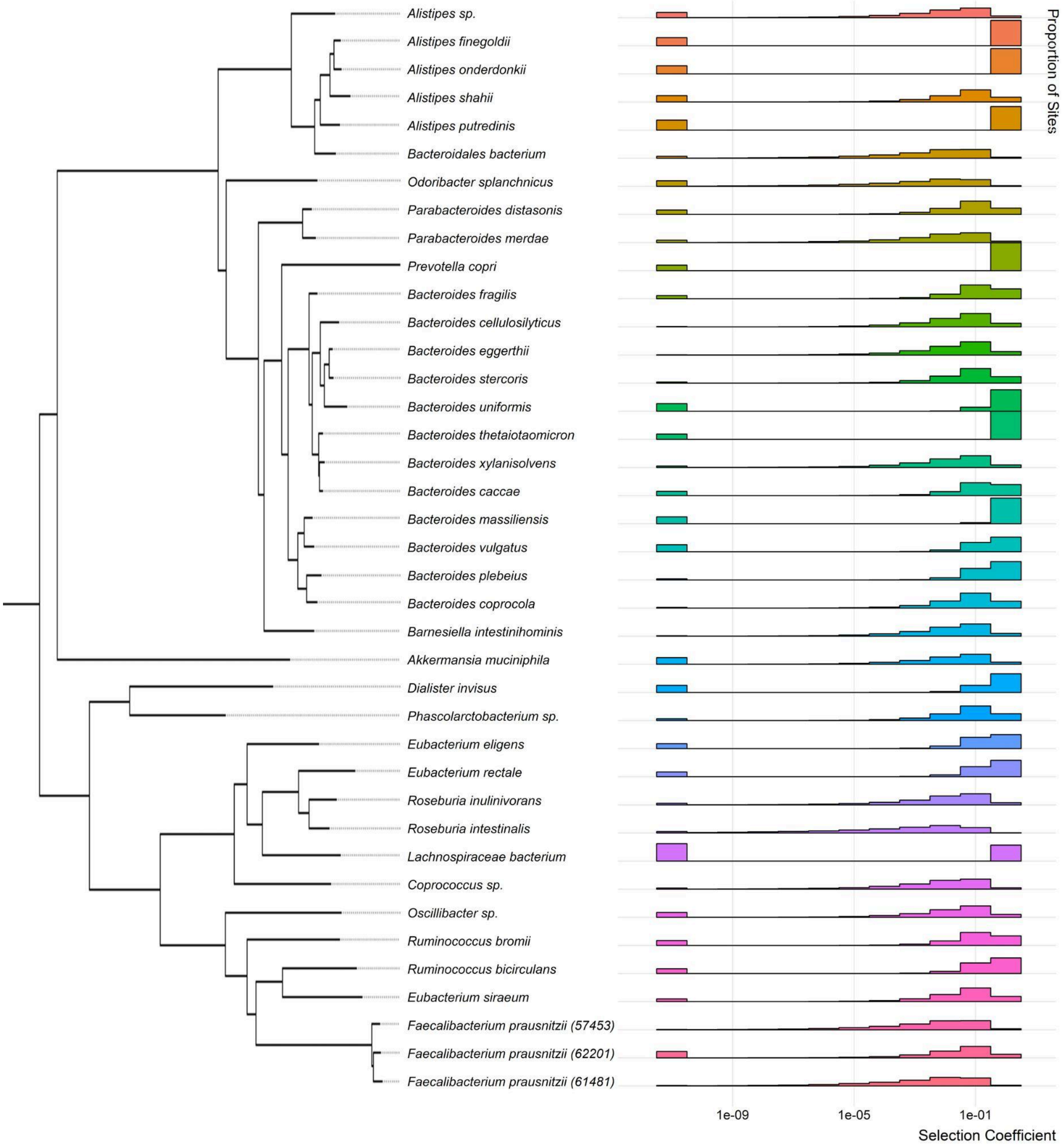

Figure S5: Neu+gamma-distributed DFE inferred from core genes
https://github.com/garudlab/microbiome_demography_manuscript/blob/main/Supplement/Supplemental_Figure_5.jpg
Phylogenetically sorted panel of inferred maximum likelihood estimates of the distributions of fitness effects per species under a neu+gamma-distribution. For each species, the x-axis denotes the $\log_{10}$ scaled discrete bin of selective effect, while the y-axis denotes the proportion of sites which fall into each bin. For ease of visualization, all mutations with selection coefficient, s, less than 1×10^{-12} are binned in the lowest bin, while all mutations with $s > 0.5$ are binned in the highest bin.

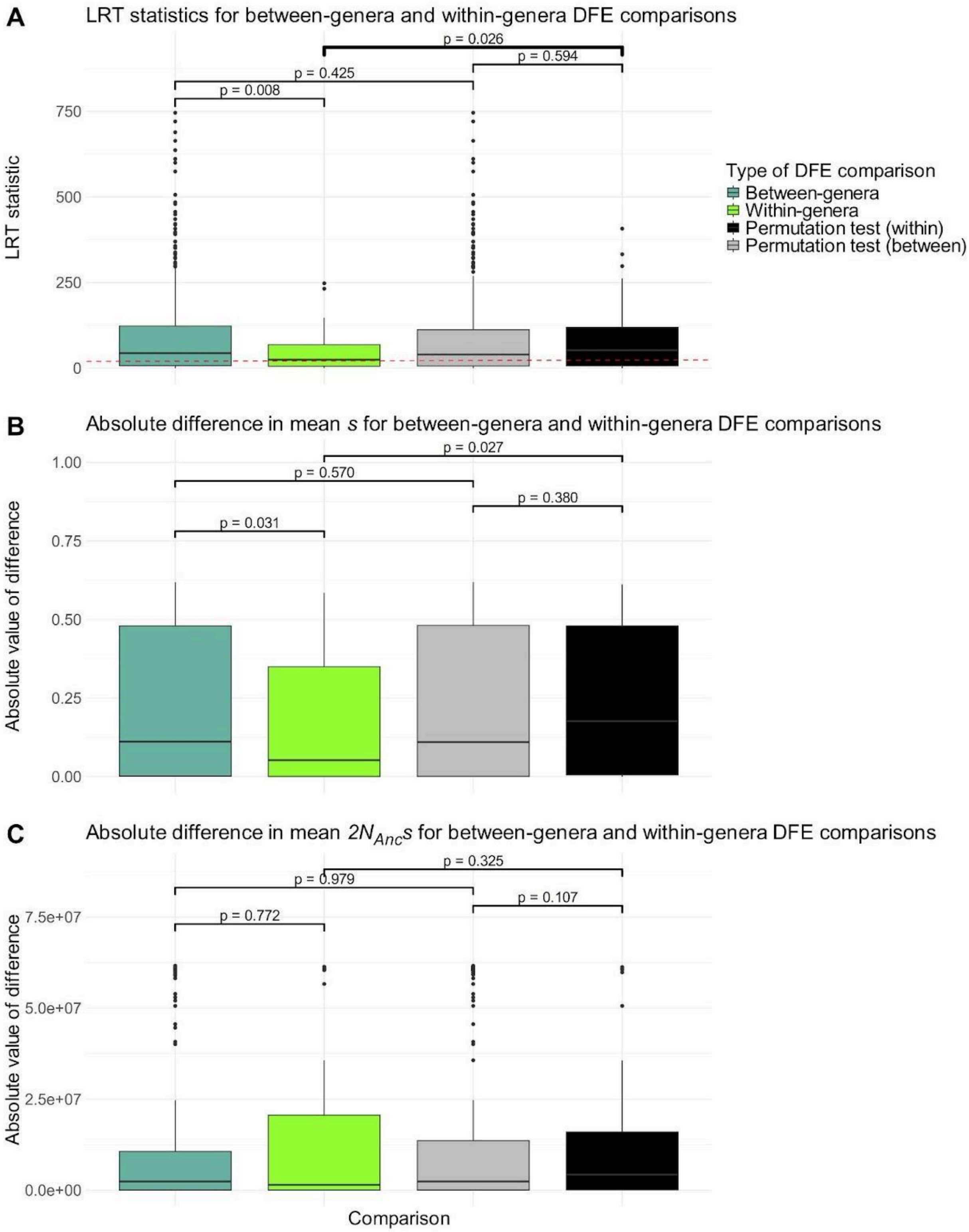
A
LRT statistics for between-genera and within-genera DFE comparisons
p = 0.026
p = 0.594
p = 0.425
p = 0.008
750
500
250
0
LRT statistic
Type of DFE comparison
Between-genera
Within-genera
Permutation test (within)
Permutation test (between)
B
Absolute difference in mean s for between-genera and within-genera DFE comparisons
p = 0.027
p = 0.570
p = 0.380
p = 0.031
1.00
0.75
0.50
0.25
0.00
Absolute value of difference
C
Absolute difference in mean 2N_Anc s for between-genera and within-genera DFE comparisons
p = 0.325
p = 0.979
p = 0.107
p = 0.772
7.5e+07
5.0e+07
2.5e+07
0.0e+00
Absolute value of difference
Comparison

Figure S6: Distributions of statistics comparing within and between-genera DFEs.
https://github.com/garudlab/microbiome_demography_manuscript/blob/main/Supplement/Supplemental_Figure_6.jpg
(**A**) Distributions of LRT statistics comparing pairs of DFEs, either from two species belonging to the same genera, or from two species belonging to two different genera. The dashed red line indicates an LRT value of 18.30, representing the critical value for statistically different DFEs. (**B**) Absolute difference in E[s] when comparing pairs of DFEs from species belonging to the same genera or from different genera. (**C**) Absolute difference in E[$2N_{\mathrm{Anc}}s$] when comparing DFEs from species belonging to the same genera or from different genera. To statistically test for differences between distributions, we performed two-sided Wilcoxon rank-sum tests. Additionally, for each of the comparisons, we permuted labels of the species 1000 times, thereby shuffling the within vs between genera status.

Figure S7: Likelihood ratio test comparing pairs of DFEs in 39 species (see attachment)
https://github.com/garudlab/microbiome_demography_manuscript/blob/main/Supplement/Supplemental_Figure_7.jpg
Each element of the matrix is color coded with a log ratio test statistic comparing a model in which pairs of species have independently inferred DFEs versus a model in which pairs of species have the same DFE. This test was performed in two ways: (**A**) evaluating the selection coefficient, s, and (**B**) evaluating the population-scaled selection coefficient $2N_{\mathrm{Anc}}s$. A likelihood ratio statistic of about 18.30 corresponds to a 95% confidence interval after Bonferroni correction for 471 tests, assuming that the LRT statistic follows a chi-squared distribution with two degrees of freedom under the null hypothesis.

Figure S8: SFSs and likelihood surfaces for demographic inference from accessory genes (see attachment)
https://github.com/garudlab/microbiome_demography_manuscript/blob/main/Supplement/Supplemental_Figure_8.jpg
(Left) Site frequency spectra from the empirical data compared to those predicted from the best-fitting models for accessory genes from 3927 common commensal gut microbiota. “MLE synonymous” shows the expected SFS produced by the ML demographic parameter estimates. “MLE nonsynonymous” shows the expected SFS produced by the ML demographic and selection parameters from a gamma-distributed DFE. (Right) 2-dimensional likelihood surfaces of population size, ν (in units of N_{Anc}) and time since the onset of the most recent demographic event, τ (in units of $\frac{generations}{2N_{Anc}}$) are shown for accessory genes from the same 3927 species for which we fit demography. A dashed red line separates the parameter space between contractions and expansions. The maximum-likelihood demographic parameter estimates are shown with an orange dot, and colored intervals denote the decrease in log-likelihood from the MLE. The light cyan regions (LL - 3) correspond to the asymptotic 95% confidence interval, from the chi-squared distribution with 2 degrees of freedom. By visual inspection, demographic inference over accessory genes yields well-fitting models in a minority (n=18) of species.

Figure S9: Empirical SFS of core and accessory genes (see attachment)

https://github.com/garudlab/microbiome_demography_manuscript/blob/main/Supplement/Supplemental_Figure_9.jpg

Empirical site frequency spectra for synonymous and nonsynonymous mutations for core genes vs accessory genes. The SFS of accessory genes display a trend of depleted rare variant frequency and increased common variant frequency compared to the SFS of core genes. Additionally, the SFS of accessory genes is much more jagged and less monotonic than the SFS of core genes.

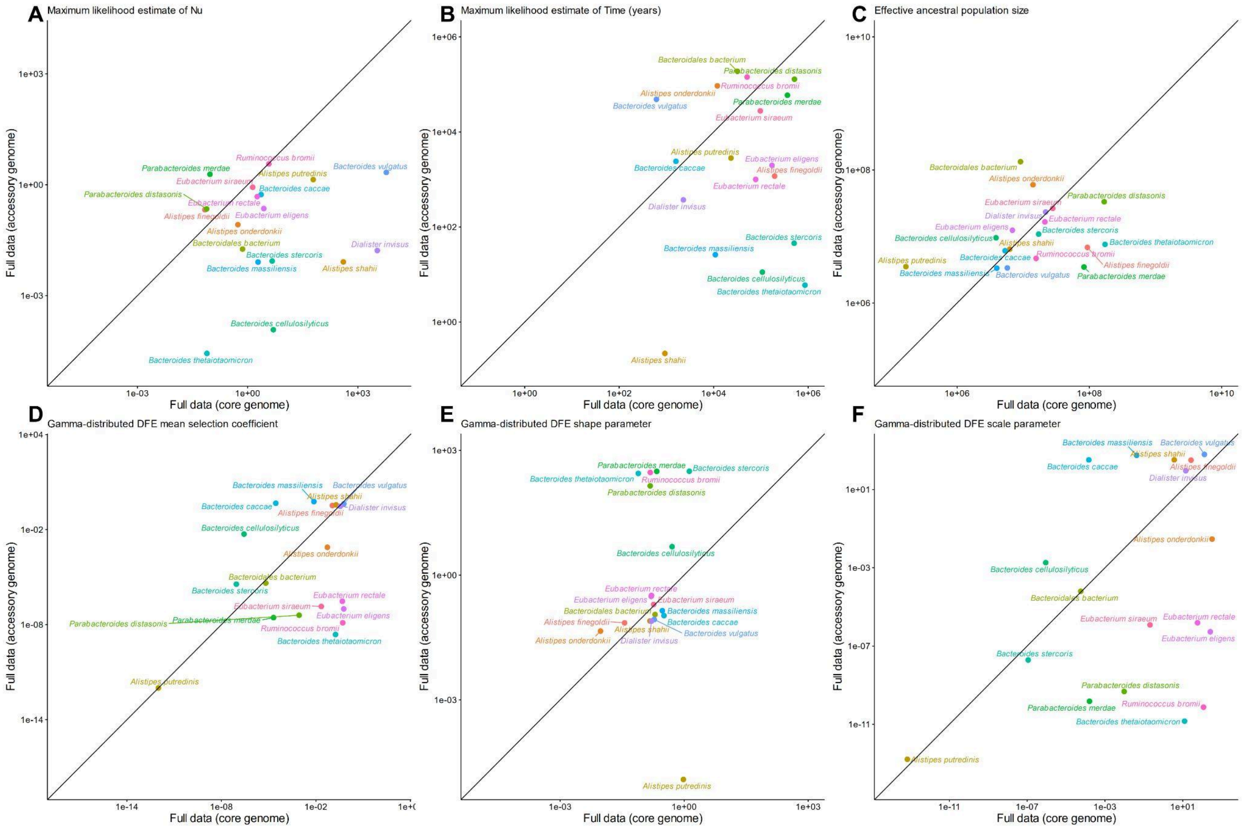

Figure S10 Comparison of evolutionary parameters inferred from the core genes (X-axis) versus accessory genes (Y-axis). The solid black-line represents the $y=x$ trend for each panel. (A) MLE of ν; (B) MLE of Time; (C) MLE of the ancestral effective population size; (D) MLE of the mean selection coefficient of a Gamma-distributed DFE; (E) MLE of the *Shape* parameter of Gamma-distributed DFE; (F) MLE of the *Scale* parameter of Gamma-distributed DFE.

Figure S11: Phylogenetically ordered analysis of core and accessory gene DFE (see attachment)

https://github.com/garudlab/microbiome_demography_manuscript/blob/main/Supplement/Supplemental_Figure_11.jpg

Comparison of the inferred gamma-distributed DFE from core genes and accessory genes for eighteen species: *Alistipes putredinis, Alistipes fragilis, Alistipes onderdonkii, Alistipes shahii, Bacteroidales bacterium, Parabacteroides distasonis, Parabacteroides merdae, Bacteroides cellulosilyticus, Bacteroides stercoris, Bacteroides thetaiotaomicron, Bacteroides caccae, Bacteroides massiliensis, Bacteroides vulgatus, Dialister invisus, Eubacterium eligens, Eubacterium rectale, Eubacterium siraeum, and Ruminococcus bromii.* . For each species, the x-axis denotes the $\log_{10}$ scaled discrete bin of selective effect, while the y-axis denotes the proportion of nonsynonymous mutations which fall into each bin. Bins are color coded from gray to dark red according to their lethality. Species for which we rejected a null hypothesis of DFE similarity between core and accessory genes are highlighted in red.

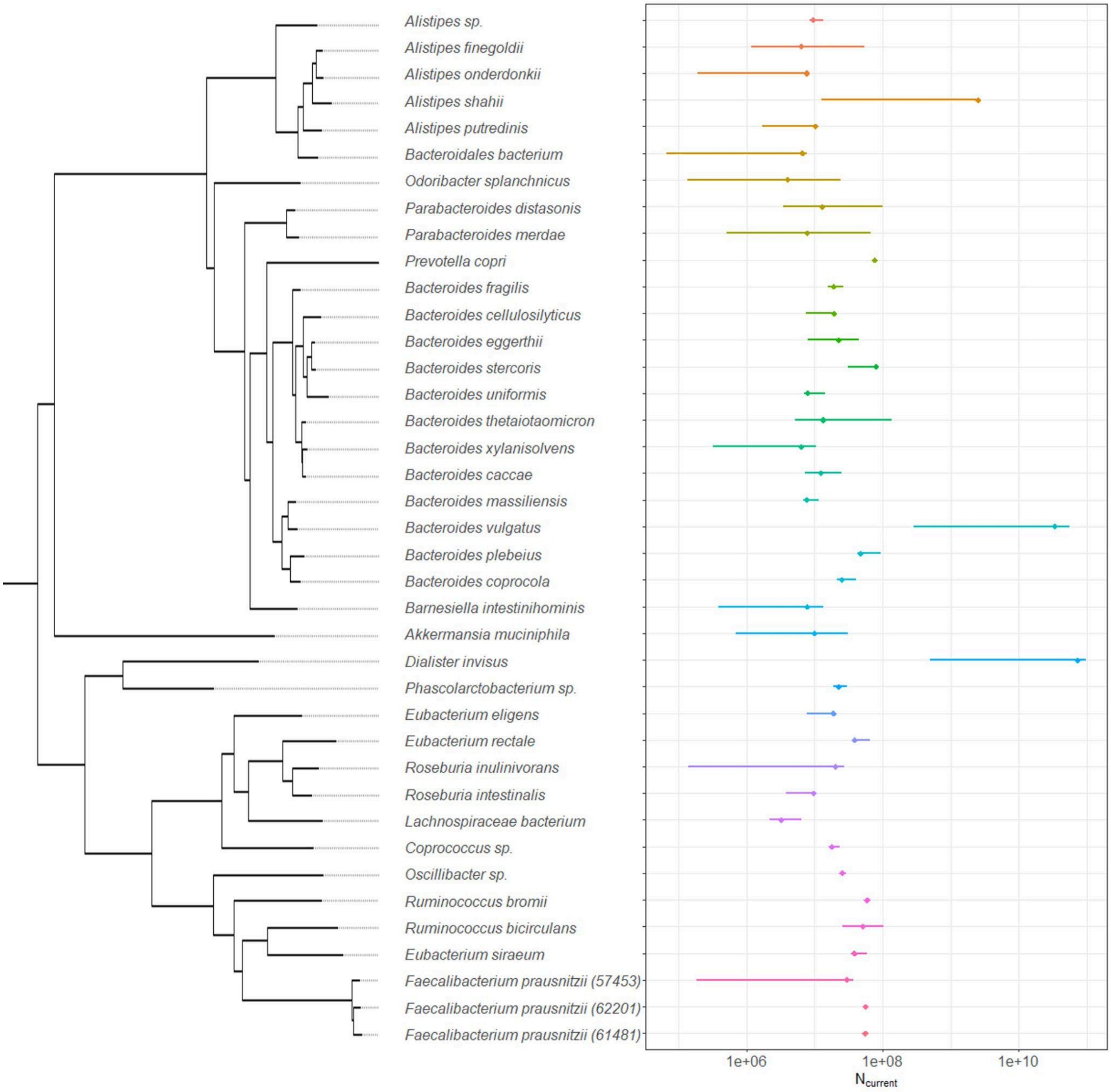

Figure S12: Distribution of N_{Curr} for core genes

https://github.com/garudlab/microbiome_demography_manuscript/blob/main/Supplement/Supplemental_Figure_12.jpg

Phylogenetically sorted panel of 95% confidence intervals N_{Curr}. The maximum likelihood parameter estimate is indicated with a diamond. Confidence intervals are color coded by species using the same color scheme as **Figure 3**.
