# Supplementary figures and images for: Inference of the Demographic Histories and Selective Effects of Human Gut Commensal Microbiota Over the Course of Human History
Source: Mol Biol Evol. 2025 Jan 22;42(2):msaf010. doi: 10.1093/molbev/msaf010 (PMC11824422; doi:10.1093/molbev/msaf010)

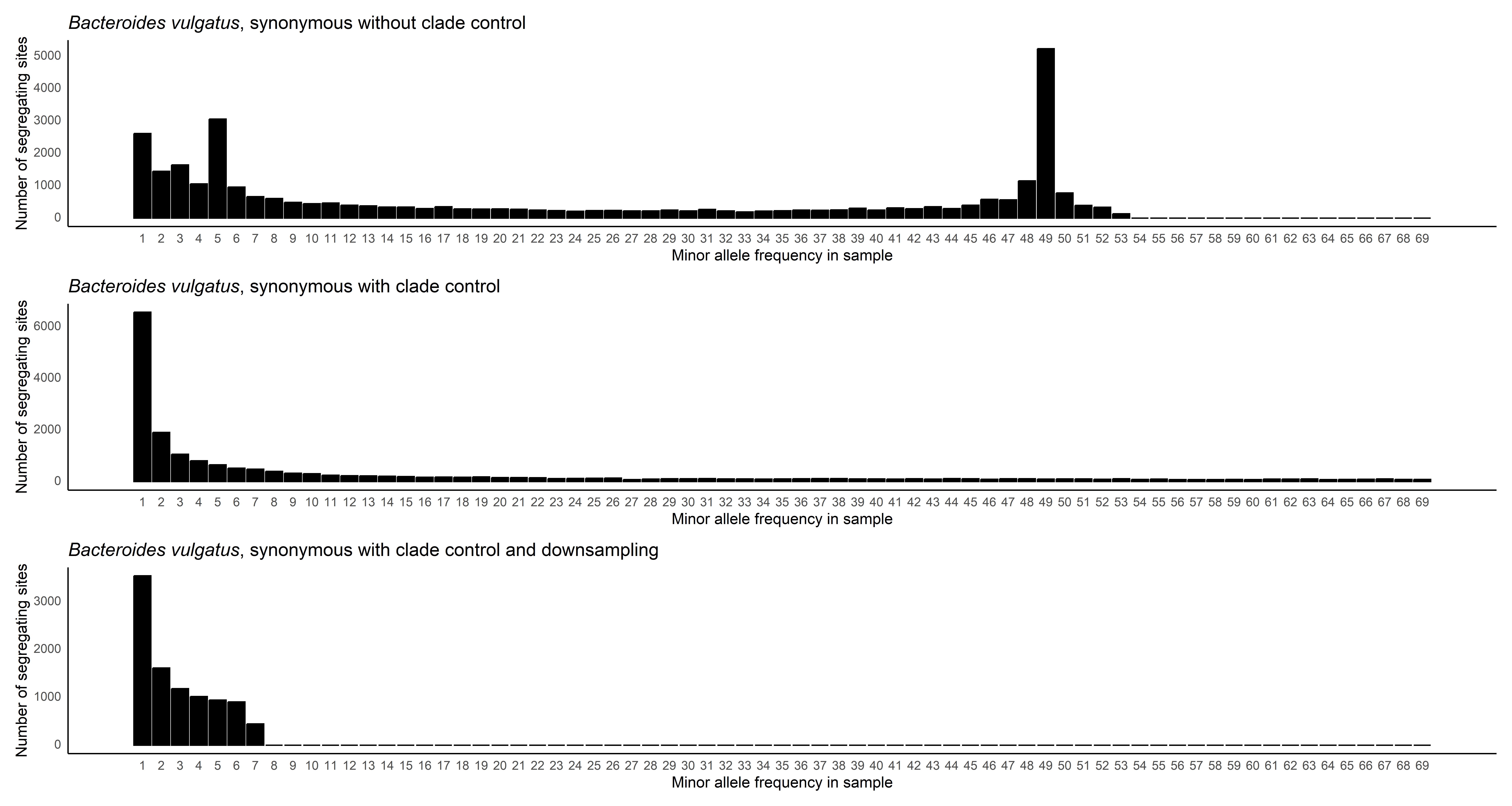

Supplement: msaf010_Supplementary_Data [file msaf010_supplementary_data.zip › Supplemental_Figure_1.jpg]

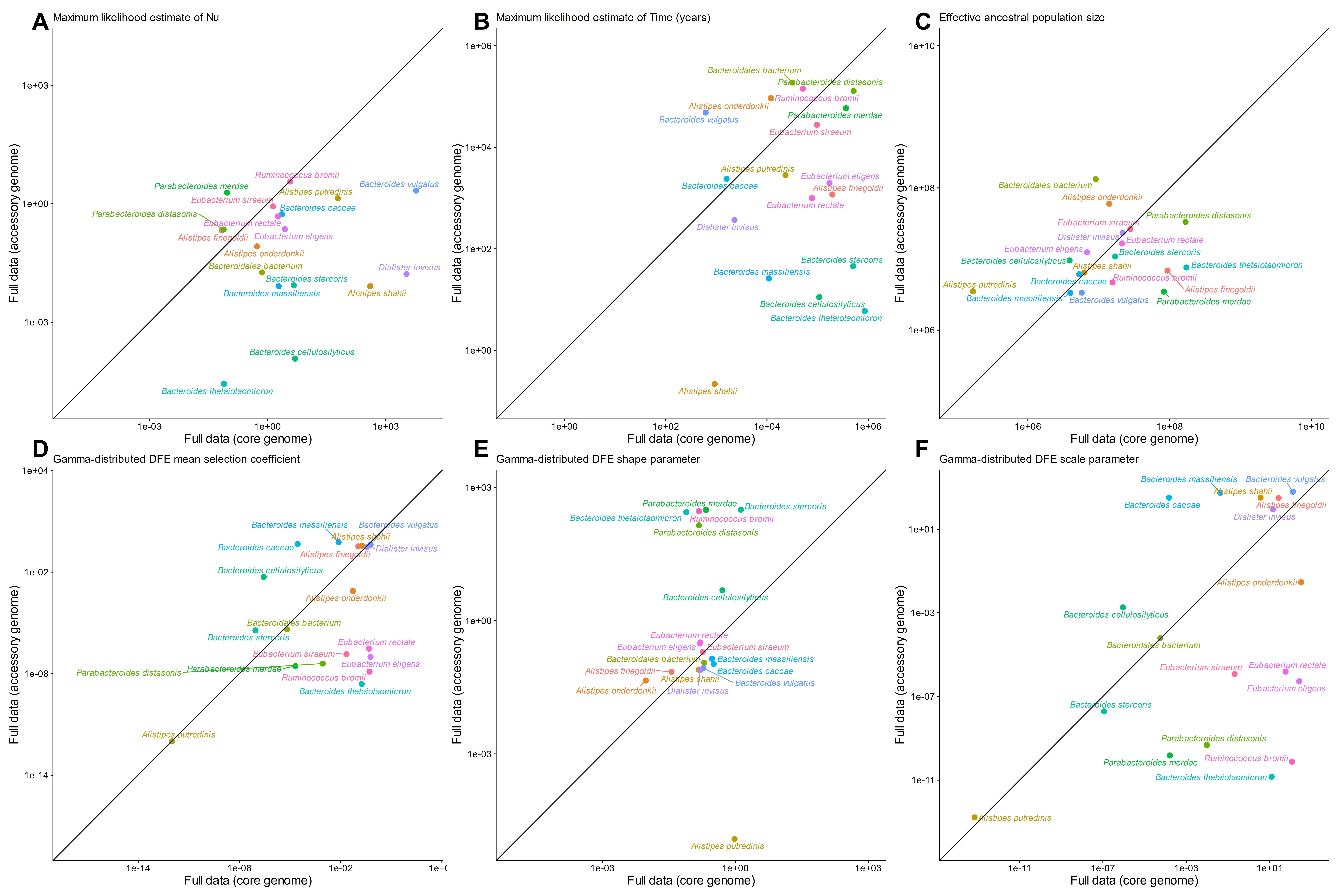

Supplement: msaf010_Supplementary_Data [file msaf010_supplementary_data.zip › Supplemental_Figure_10.jpg]

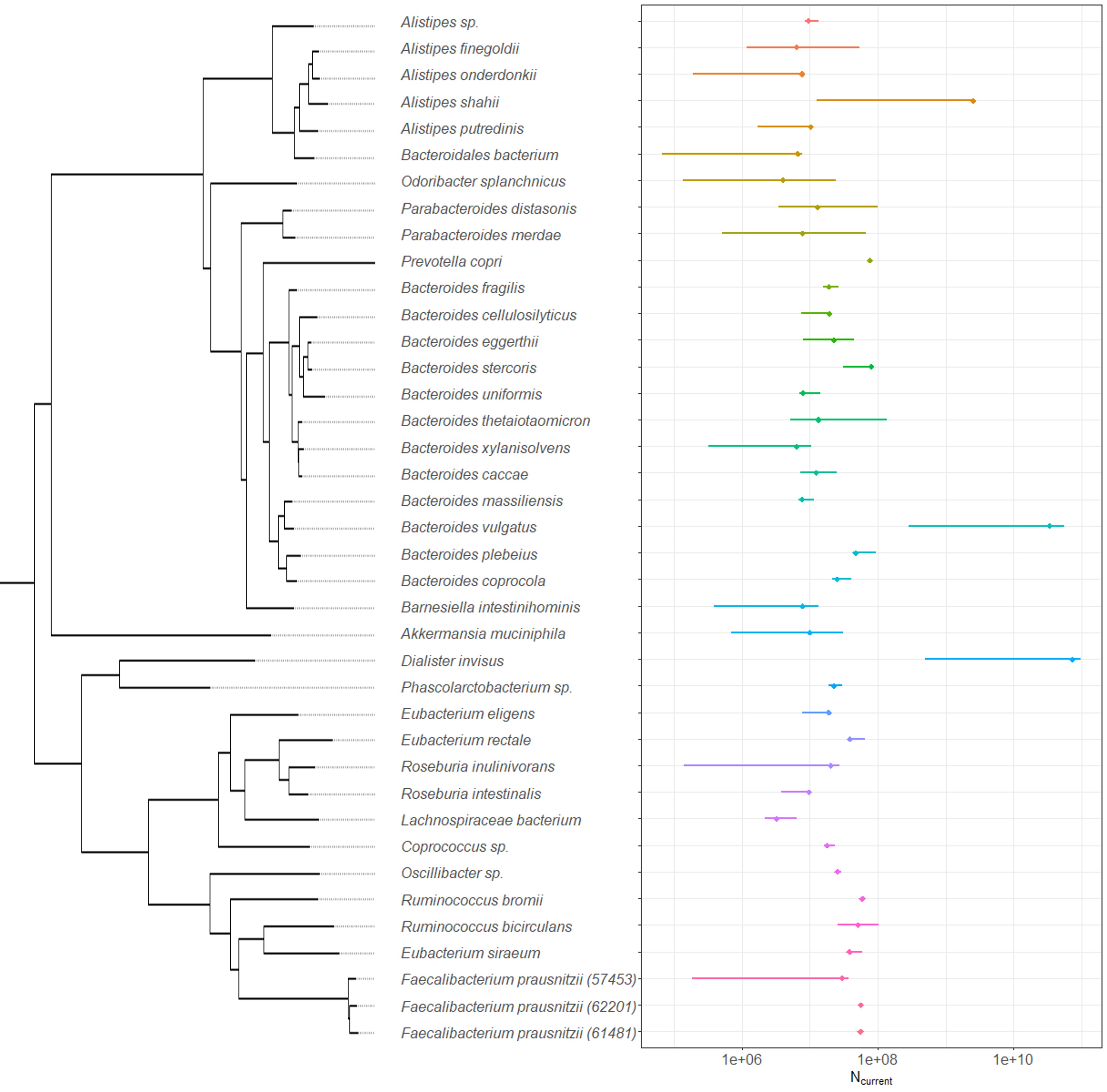

Supplement: msaf010_Supplementary_Data [file msaf010_supplementary_data.zip › Supplemental_Figure_12.jpg]

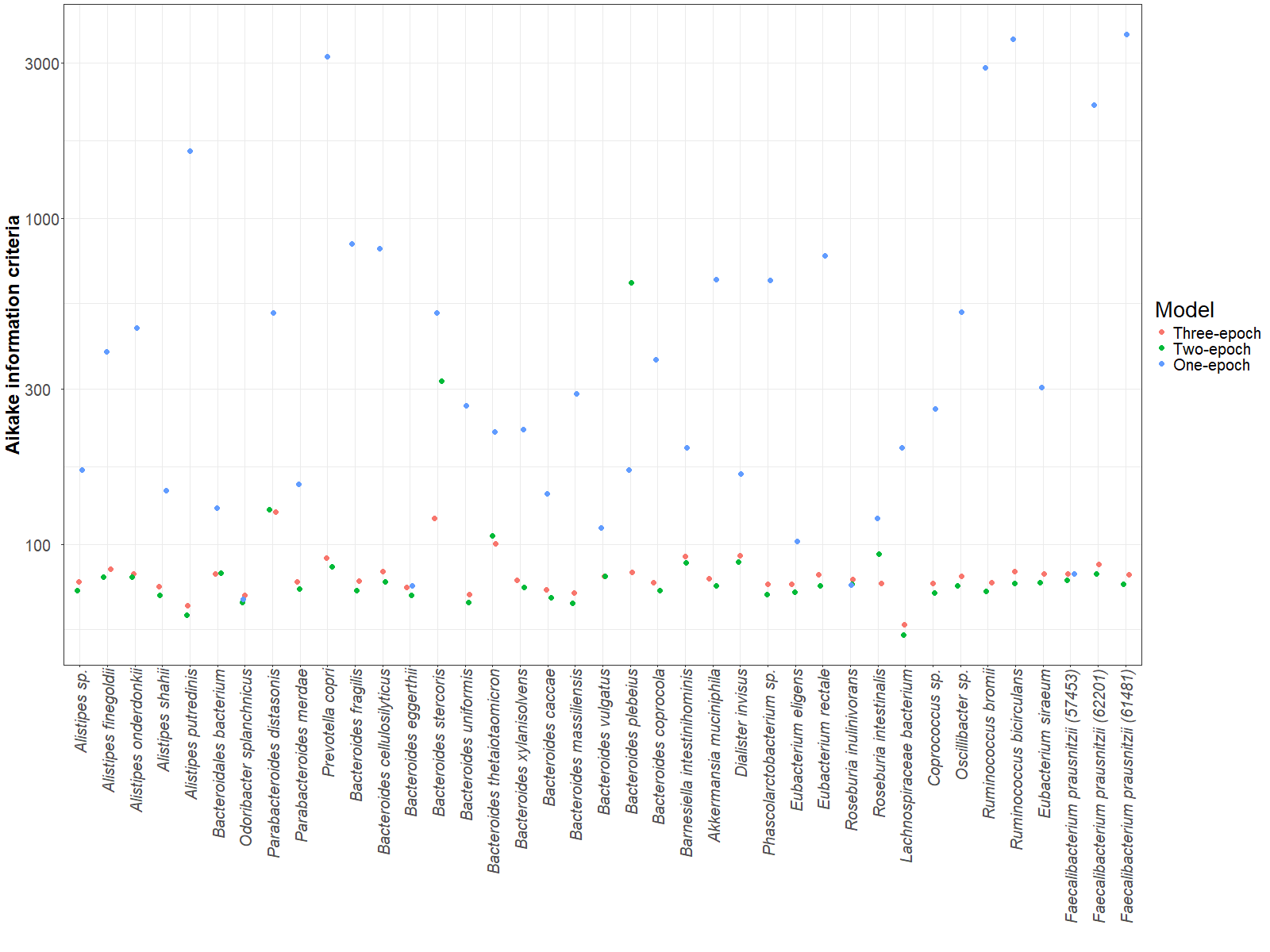

Supplement: msaf010_Supplementary_Data [file msaf010_supplementary_data.zip › Supplemental_Figure_2.jpg]

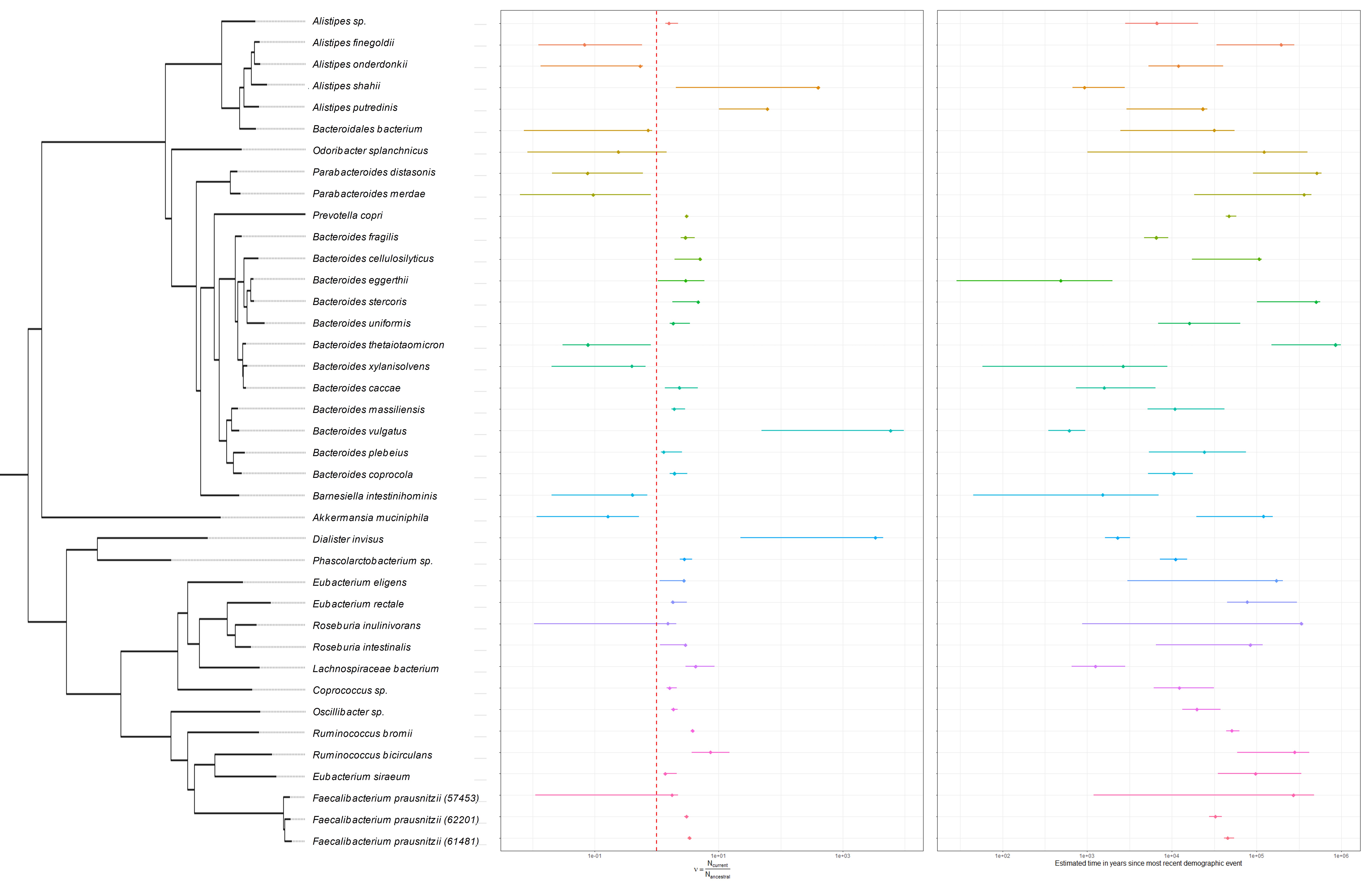

Supplement: msaf010_Supplementary_Data [file msaf010_supplementary_data.zip › Supplemental_Figure_4.jpg]

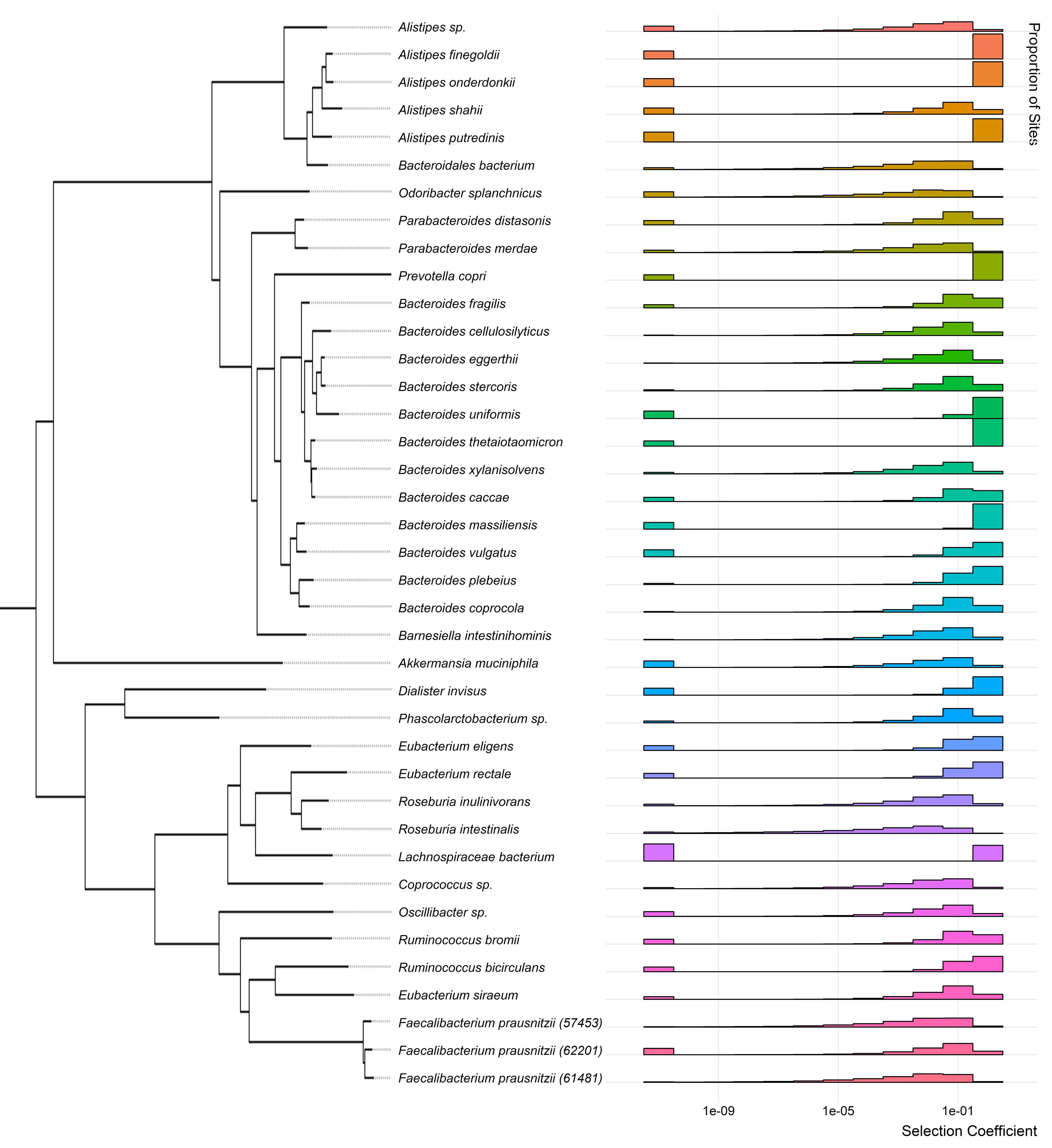

Supplement: msaf010_Supplementary_Data [file msaf010_supplementary_data.zip › Supplemental_Figure_5.jpg]

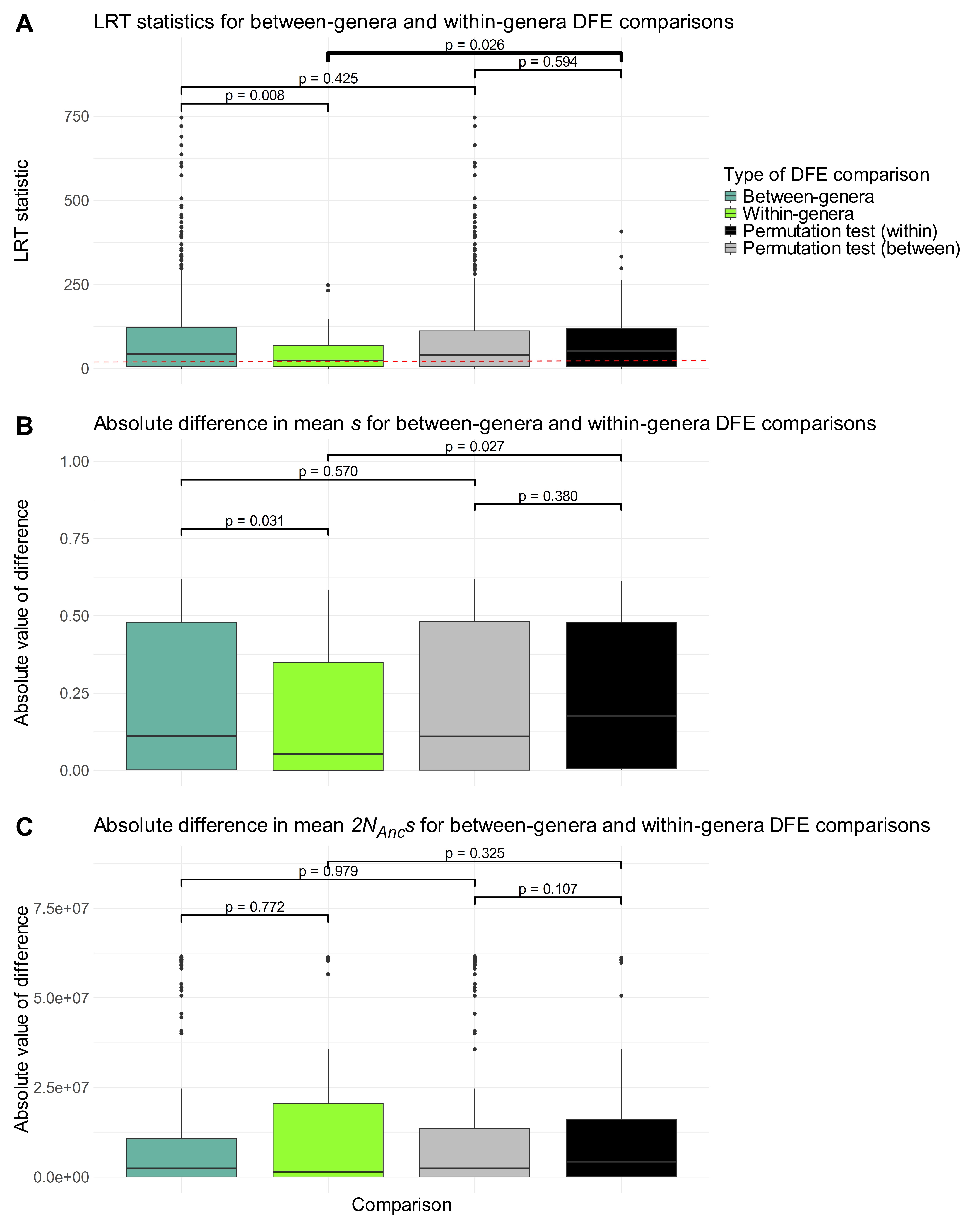

Supplement: msaf010_Supplementary_Data [file msaf010_supplementary_data.zip › Supplemental_Figure_6.jpg]

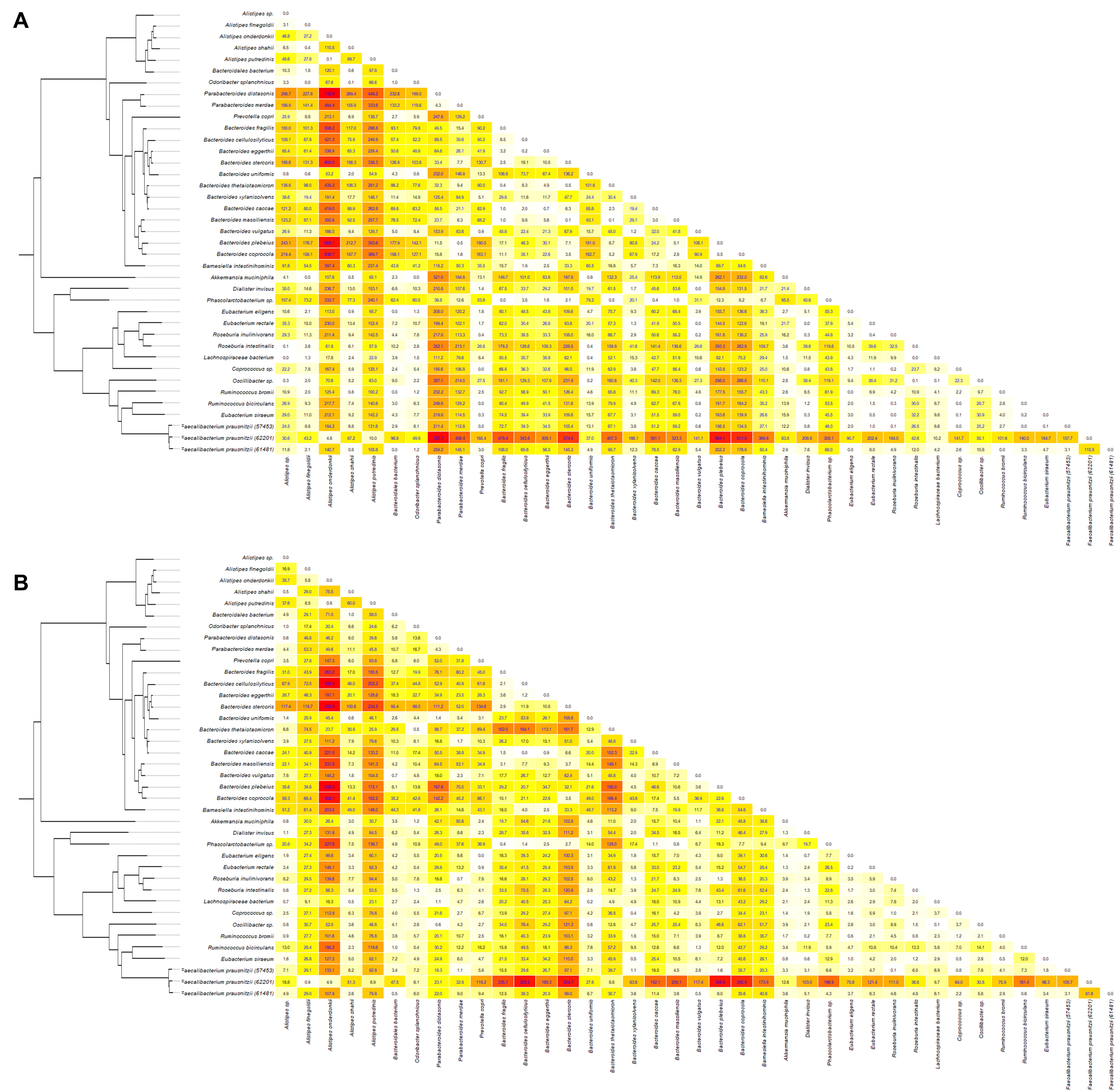

Supplement: msaf010_Supplementary_Data [file msaf010_supplementary_data.zip › Supplemental_Figure_7.jpg]
